# The OAS1-FASN axis promotes pancreatic cancer by coordinating lipogenic stress and the unfolded protein response

**DOI:** 10.1038/s41419-026-08922-8

**Published:** 2026-06-03

**Authors:** Yuheng Zhu, Hongfei Yao, Chunjing Li, Jie Peng, Jieqiong Ge, Meng Liu, Tongyi Zhang, Zhiwei Cai, Chongyi Jiang

**Affiliations:** 1 Department of Hepato‑Biliary‑Pancreatic Surgery, General Surgery, Huadong Hospital, Fudan University, Shanghai, PR China; 2 Department of Gastrointestinal Surgery, Ren Ji Hospital, School of Medicine, Shanghai Jiao Tong University, Shanghai, PR China; 3 Department of Nursing, Huadong Hospital, Fudan University, Shanghai, PR China; 4 Shanghai Key Laboratory of Clinical Geriatric Medicine, Shanghai, PR China

**Keywords:** Cancer metabolism, Pancreatic cancer

## Abstract

Metabolic reprogramming, characterized by dysregulated lipid metabolism and consequent endoplasmic reticulum (ER) stress, constitutes a hallmark of pancreatic ductal adenocarcinoma (PDAC). Within this metabolic landscape, the 2′-5′-Oligoadenylate Synthetase (OAS) family member OAS1 is identified as a critical driver of malignancy, exhibiting specific upregulation in PDAC tissues that correlates with poor patient prognosis. Functionally, OAS1 drives tumor progression, including cell proliferation and metastasis, by operating as a non-canonical metabolic regulator. Mechanistically, OAS1 binds to fatty acid synthase (FASN), maintaining its functional protein levels and thereby promoting FASN-dependent lipid synthesis. The resulting surge in de novo lipid synthesis and lipid droplet accumulation precipitates an adaptive ER stress response via the PERK-ATF4 signaling axis. Consequently, a functional OAS1-FASN axis operates to coordinate lipid overload with pro-survival ER stress signaling, establishing OAS1 as a pivotal metabolic regulator and a viable biomarker in PDAC.

**Figure:**
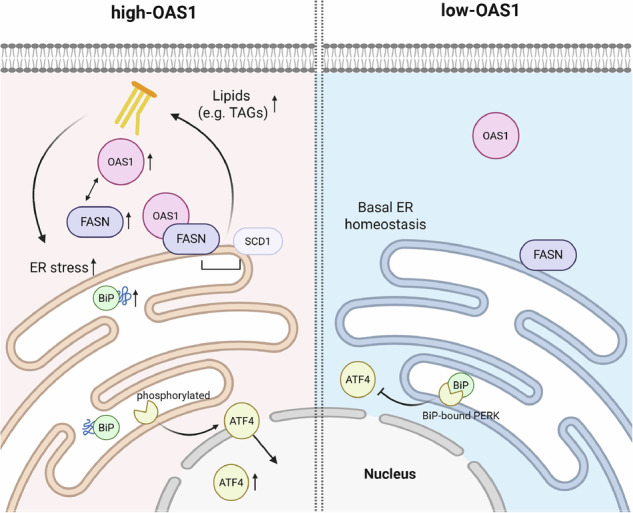

## Introduction

Pancreatic ductal adenocarcinoma (PDAC) remains a formidable clinical challenge, characterized by its aggressive biology and resistance to current therapies [1, 2]. Within a microenvironment widely known as dense, immunosuppressive, metabolite-disordered, PDAC tumor cells usually exhibit highly dynamic adaptability and metabolic reprogramming [3–9]. Notably, research has been continuously revealing the important role of lipid metabolism reprogramming in the malignant biological behaviors of PDAC [10–13]. Therefore, it’s necessary to further study the mechanisms of lipid metabolism in PDAC for identifying novel biomarkers and developing effective therapeutic strategies.

In PDAC, lipid metabolism, including fatty-acid synthesis and uptake, is among the central nutrient metabolism pathways, which are frequently reprogrammed to meet constant stress. These lipids supply membranes, energy, and signaling of tumor cells [14, 15]. This heightened metabolic activity places a substantial burden on the endoplasmic reticulum (ER), the primary site of lipid synthesis. Consequently, PDAC cells tend to exhibit chronic ER stress, activating the unfolded protein response (UPR) to maintain homeostasis [16, 17]. Shifts in lipid composition, such as an increase in saturated fatty acids, can disrupt ER membrane integrity and function, further triggering UPR signaling through sensors like BiP and the nuclear translocation of ATF4 [17–20]. While this interplay between lipid overload and ER stress is known to support tumor progression, the specific molecular regulators that participate upstream in this process remain to be explored.

By screening the expression of fatty acid metabolism (FAM)-related genes by signature in public databases, a group of genes, the 2′-5′-oligoadenylate synthetase family with OAS1, OAS2, OAS3, and OASL as four members, are identified as potential candidates for the regulators of lipid metabolism in several types of tumors, with OAS1 especially outstanding in PDAC. The OAS family members are generally recognized as interferon-stimulated genes (ISGs) for interferon signaling and antiviral defense, but their roles in tumor biology are now drawing more attention [21, 22]. Previous studies have connected OAS1 with patient prognosis in several cancers, and PDAC cohorts also suggest higher OAS1 in tumors with poorer prognosis [23–27]. Recently, OASL has been reported to reshape lipid programs in inflammation-related tumor cells [28]. However, whether members with enzymatic activity, such as OAS1, participate in PDAC lipid metabolism has not yet been defined mechanistically.

In this study, we show that OAS1 is the key member of the OAS family whose expression is consistently associated with adverse clinical outcomes in PDAC. The up-regulated expression of OAS1 leads to proliferation, migration, and invasion of PDAC as cellular function and tumoral progression both in vitro and in vivo. Mechanistically, our work identifies an interaction between OAS1 and fatty acid synthase (FASN). This interaction drives a high load of lipid metabolism through the IFN-independent OAS1-FASN axis with an ATF4-mediated adaptive ER stress response that promotes PDAC progression. Our findings identify OAS1 as the upstream initiator of the adaptive response axis within PDAC cells that links a classical immune-related protein to a central metabolic pathway, expanding the understanding of the non-canonical metabolic roles of OAS1 as a key member of the OAS family in cancer.

## Methods and materials

### Public datasets

RNA sequencing data with matched clinical information of pancreatic adenocarcinoma and other types of cancer were accessed and downloaded from The Cancer Genome Atlas (TCGA) data portal (https://portal.gdc.cancer.gov/repository). Samples with incomplete clinical data were artificially excluded. Normal pancreas control samples were accessed and obtained from the pancreas dataset of Genotype-Tissue Expression (GTEx) Project (https://www.gtexportal.org). In total, 178 tumor samples from the TCGA-PAAD database and 167 normal pancreas samples from the GTEx database were collected and analyzed.

GEO datasets include GSE28735, GSE62452, and GSE71729.

Single-cell datasets include GSE111672 and CRA001160.

### Bioinformatics analysis

Public datasets were obtained as described above. The FAM hallmark gene set and UPR gene set were obtained and filtered from the Molecular Signatures Database (MSigDB). Expression values of the FAM hallmark genes were extracted from the TCGA-PAAD database. The R package ConsensusClusterPlus was applied for unsupervised consensus clustering. Overall survival (OS) and disease-free survival (DFS) of the clusters were analyzed using the R packages of survival and survminer.

GEPIA2 (http://gepia2.cancer-pku.cn/) was used in correlation analysis, survival maps, expression maps of the OAS family across TCGA cohorts, and OS and DFS analyses in TCGA PAAD datasets. STRING (https://string-db.org/) was used in multiple protein analyses. BEST from Hiplot (https://rookieutopia.hiplot.com.cn/app_direct/BEST/) was employed for the expression analyses of OAS family members in the GEO datasets. OAS family distribution analyses among single-cell RNA-seq datasets were performed upon Tumor Immune Single-cell Hub 2 (TISCH2) (http://tisch.comp-genomics.org/home/).

### Clinical samples and tissue microarrays

The PDAC tissues and matched non-tumor pancreatic tissues were collected under IRB approval (2023K019 by HDIRB) from Huadong Hospital, Fudan University. The tissue microarray (TMA) contains a total of 100 pairs of primary tumor tissues and adjacent non-tumor ones, with clinical information matched to each sample. All human samples involved were collected with prior informed consent. All of the tissues have been pathologically certified. All available samples meeting the inclusion criteria were included to maximize statistical power.

### Immunohistochemistry (IHC) assay

Tissue sections underwent a continuous process of dewaxing, hydration, endogenous peroxidase blocking, epitope retrieval, 5% BSA blocking, overnight primary antibodies incubation, secondary antibodies incubation, DAB, hematoxylin staining, optional 1% hydrochloric acid alcohol differentiation, ethanol dehydration, xylene transparency, neutral resin sealing, and air drying before microscopic observation and imaging. Antibodies and dilution ratios in this assay are listed in Supplementary Table 1.

Two pathologists, blinded to the groups and clinical data, scored the stained slides and TMA. We used immunoreactive score (IRS) (intensity 0–3 × area 0–3) and histochemistry score (H-score) as H-Score = Σ (intensity × positive area percentage). For clinical correlation and survival analyses, patients were dichotomized into high and low OAS1 expression groups based on a pre-specified IRS cutoff of 4 (median value). Specifically, an IRS of 0–3 was defined as low expression, while an IRS of 4–9 was defined as high expression. The H-score was used descriptively to confirm staining consistency.

### Immunofluorescence (IF) assay

Suspended cells within specific medium were seeded onto confocal dishes, glass coverslips, or 8-well plates, cultured to 60–80% confluence before observation with confocal imaging systems. Cell samples were 4% paraformaldehyde fixed in for 15 min at room temperature, and then washed with PBS. A 10-min 0.1% Triton X-100 incubation at room temperature was performed if needed. A 1-h primary antibody incubation at 4 °C was performed after a 30-min 5% BSA blocking. After three times of PBS washes, a 1-h fluorescent secondary antibody incubation at room temperature was carried out in a dark place. DAPI was used for nuclear counterstain for 5 min, followed by a 3 × 5 min wash with PBS before imaging. The fluorescence intensity was quantified using ImageJ. For each group, at least 5 random fields (containing at least 50 cells) were captured under identical exposure settings. The total integrated density was normalized to the number of cells or the total cell area to determine the mean fluorescence intensity. The ATF4 nuclear translocation ratio was calculated as the number of cells with distinct nuclear ATF4 signals divided by the total number of DAPI-positive cells in each field. A minimum of 200 cells per experimental condition were analyzed to ensure statistical robustness.

Antibodies and dilution ratios in IF staining assays are listed in Supplementary Table 1. BODIPY was used for live cell staining for lipid droplets with BODIPY 493/503 Staining Kit (C2053M, Beyotime). Cells on plates were incubated in the prepared solution for 30 min at 37 °C, then PBS-washed and imaged immediately after DAPI staining. ER-Tracker Red (C1041S, Beyotime) was also used for live cell staining for ER observation. Cells were HBSS-washed, incubated with dye solution for 20 min in the dark at room temperature, and imaged immediately after DAPI staining. Confocal microscopes (Leica, Germany) were used in this imaging process.

### Live-cell imaging

Cells for lipid and ER imaging were seeded on specific glass-bottom confocal dishes compatible with the imaging system (SC3000, ZIRCON, China). No staining was performed when observing lipid droplets by intensity diffraction tomography, where they were shown as regular white or black tiny round dots (whose color depends on the layer), while the ER stained by ER Tracker described above was observed under TxRed fluorescence. Contrast ratio, Time-lapse settings, and exposure were kept constant across groups.

### Transmission electron microscopy

For ultrastructural analysis, cell samples undergone treatments were trypsinized, washed in PBS, and initially fixed with 2.5% glutaraldehyde overnight at 4 °C. After a buffer rinse, the samples underwent a 1.5-h post-fixation at room temperature. Next, cell samples underwent a graded ethanol series and were embedded in acetone resin overnight. The samples were then transferred to a specific laboratory with operators for the subsequent imaging with the transmission electron microscope (HITACHI HT7800, Hitachi) at an acceleration voltage of 120 kV. A CCD camera was used for image acquisition. The ER surface density was quantified using the ImageJ software. Briefly, 10 representative micrographs per group were randomly selected at a magnification of 10,000×. The total area of the cytoplasm and the total length or surface area of the ER membranes were measured. The ER surface density was calculated as the ratio of ER membrane area to the cytoplasmic area, providing a standardized measure of ER abundance and structural integrity.

### Cell culture and reagent treatments

The human PDAC cell lines (AsPC-1, BXPC3, CFPAC, Mia PaCa-2, PAC-1, Patu-8988, PDC0034, SW1990), hTERT-Human Pancreatic Nestin-Expressing (HPNE), and HEK293T used in this research were either preserved in Shanghai Cancer Institution or obtained from the Cell Bank of the Chinese Academy of Sciences (Shanghai, China). KPC-1199 stably expressing firefly luciferase (KPC-1199-Luc) was from Shanghai Cancer Institution. All cells in this study were maintained in a regularly sterilized, humidified incubator at 37 °C with 5% CO₂. The culture medium for each cell line was based on ATCC recommendations, with 10% fetal bovine serum, 1% penicillin/streptomycin, and mycoplasma elimination reagent (40607ES03, YEASON) to ensure regular mycoplasma test results were negative.

ER stress inducer Tunicamycin (2.5 μM for 24 h; HY-A0098, MedChemExpress), ER pathways inhibitors ISRIB (0.5 μM for 24 h; HY-12495A, MedChemExpress), 4μ8C (50 μM for 24 h; HY-19707, MedChemExpress), and Ceapin-A7 (2 μM for 24 h; HY-108434, MedChemExpress) were used as pharmacological interventions of ER stress. TVB-2640 (Denifanstat; HY-112829, MedChemExpress), a potent and selective FASN inhibitor, was dissolved in DMSO to create a stock solution. For IC_50_ determination, cells were treated with a range of concentrations (0.005–100 μM) for 72 h, and cell viability was assessed via CCK-8 assay (five replicates per group). For functional rescue and phenotypic assays, cells were treated with TVB-2640 at indicated concentrations (Patu-8988, 0.3 μM; PDC0034, 1.0 μM; 0–50 μM for FASN functional output measurement in FASN-rescue experiments) according to IC_50_ (triplicate per group). DMSO was used as the vehicle control in all experiments. Human IFN-alpha A (alpha 2a) Recombinant Protein (1000 U/mL for 24 h; PHC4014, Thermo Fisher Scientific) and anti-IFNAR2 (10 μg/mL for 1 h before IFN; 213851, Thermo Fisher Scientific) were used for interferon signaling verification.

### Cellular FASN functional assays

The functional output of FASN in PDAC cell lysates was evaluated using a FAS Activity Assay Kit (BC0550, Solarbio) according to the manufacturer’s instructions (triplicate per group). Briefly, 5 × 10^6^ cells were harvested and lysed in 1 mL of extraction buffer using an ice-cold ultrasonic cell disruptor. The supernatant was collected after centrifugation at 12,000 × *g* for 20 min at 4 °C. The reaction was initiated by adding the cell extract to a mixture containing acetyl-CoA, malonyl-CoA, and NADPH. The rate of decrease in absorbance at 340 nm was monitored at 37 °C using a microplate reader, representing the consumption of NADPH during long-chain fatty acid synthesis. One unit of FASN functional output (U) was defined as the amount of functional FASN protein that oxidizes 1nmol of NADPH per minute. Specific activity was calculated using the following formula:

FASN functional output (U/10^4^ cells) = 1607.5 × Δ*A* ÷ *N*

Δ*A* is the change in absorbance per minute, and *N* is the number of cells counted in 10^4^.

### Knockdown and overexpression assays

Lentiviruses with negative control or OAS1-targeting short hairpin RNA (shRNA) sequences and small interfering RNAs (siRNAs) targeting ATF4 were purchased from OBiO Technology (Shanghai, China). The pSLenti-pA-MCS-CMV-EF1-Puro-WPRE lentivirus was used as a control vector, and recombinant lentiviruses expressing FASN were constructed for overexpression assays. For siRNA transfection, PDAC cells were seeded into 6-well plates and transfected at a 70% confluence using Lipofectamine 3000 (L3000001, Invitrogen). For lentivirus transfection, transductions were performed with 1:200 polybrene, and stable clones were screened and selected with 5 μg/mL puromycin. Sequences are listed in Supplementary Table 2.

### Cell proliferation assessing assays

Cell proliferation ability was estimated using Cell Counting Kit-8 (ShareBio, SB-CCK8). Briefly, PDAC cells were seeded into 96-well plates at 1500 cells/well (five replicates per group). Every 24-h post-seeding, absorbance at 450 nm was measured by an enzyme-linked immune detector (M1000 PRO, Tecan) for 4–5 days after incubation with 10% CCK-8 medium for 2 h at 37 °C.

The colony formation assay was also for cell proliferation ability assessing. PDAC cells were seeded into 6-well plates at 1500 cells/well (triplicate per group), optionally treated, and cultured for 14 days. After washing, cells were fixed in 4% paraformaldehyde for 15 min, washed, stained with 0.1% crystal violet (G1014, Servicebio), imaged, and then analyzed. Three independent experiments were performed in these assays.

### Cell invasion and migration assessing assays

For Transwell assays, 300 μL serum-free medium with 5 × 10⁴ suspended PDAC cells were gently injected into the upper chambers of 24-well Transwell plates (Corning, USA). The lower chambers contained 700 μL medium with 10% FBS. After a 36-h culture at 37 °C, contents in the upper chamber were removed. The cells on the lower membrane surface were fixed in 4% paraformaldehyde, and then immediately stained by 0.1% crystal violet. Images of three different fields of each chamber membrane were captured at 100× magnification for each group using a Zeiss AXIOVERT 200 microscope (Zeiss, Germany) and analyzed.

For wound-healing assays, confluent monolayers of PDAC cells within 6-well plates were scratched by sterile 200 μL tips. Wound closure was imaged at timepoints of 0 h, 12 h, and 24 h at 100× magnification under a Zeiss AXIOVERT 200 microscope (Zeiss, Germany). Migration rates were calculated from three random fields per well, triplicate per group. Three independent experiments were performed in these assays.

### Quantitative real-time polymerase chain reaction (qRT-PCR) assay

Total RNA of cells was extracted using RNAiso Plus (9109, Takara Bio). Next, reverse transcription was performed with the PrimeScript™ RT Reagent Kit (RR037A, Takara Bio). After measurement of RNA concentration, qRT-PCR was performed using TB Green Premix Ex Taq™ Ⅱ (RR820A, Takara Bio) with the Applied Biosystems™ 7500 Real-Time PCR System (Thermo Fisher Scientific, USA). The amplification program was 95 °C for 10 min, followed by 40 cycles of 95 °C for 10 s, and 60 °C for 30 s. 18S level was detected and used for normalization. The sequences of primers used are listed in Supplementary Table 2.

### Western blotting

Cell protein was extracted using the protease and phosphatase inhibitors, mixed RIPA Lysis and Extraction Buffer (89900, Thermo Fisher Scientific), and concentrations were tested with a BCA Protein Assay Kit (SB-WB013, ShareBio). Protein samples of equal amounts were separated in a 7.5 or 10% gel by SDS-PAGE and transferred onto nitrocellulose membranes. The membranes then underwent TBST washes, 5% skim milk blocking, cutting, and incubation with target-specific primary antibodies at 4 °C overnight with gentle shaking. After three times of TBST washes, species-specific secondary antibodies were used to incubate the membranes for 1 h with gentle shaking at room temperature. Protein bands on the membranes were visualized with the ChemiDoc XRS+ chemiluminescence imaging system (Bio-Rad, USA) and quantified by densitometry. Antibodies and dilution ratios in Western blotting are listed in Supplementary Table 1. Original, uncropped images for all immunoblots presented in this study are provided in the Supplementary Material.

### RNA sequencing

Total RNA from shNC- and shOAS1-transfected PDAC cells was extracted as described above, and RNA integrity was confirmed using an Agilent 5300 Bioanalyzer (Agilent Technologies). Sequencing was performed by Sinotech Genomics (Shanghai, China) according to standard protocols. Briefly, mRNA was purified, fragmented, and converted into libraries, which were clustered by the TruSeq PE Cluster Kit v3-cBot-HS (Illumina). Fragments per kilobase per million reads (FPKM) was used for gene expression quantification. Analyses for pathway enrichment (Kyoto Encyclopedia of Genes and Genomes (KEGG) and Gene Ontology (GO)) and other follow-up analyses were based on differentially expressed genes (DEGs).

### Untargeted metabolomics

Untargeted metabolomics were performed by Sinotech Genomics (Shanghai, China). For sample preparation, cells were rinsed with ice-cold PBS to halt metabolic activity. Metabolites were then extracted using a cold methanol, acetonitrile, and water solution that was supplemented with internal standards. After protein precipitation and centrifugation, supernatants were dried and reconstituted for liquid chromatography-mass spectrometry (LC-MS). Chromatographic separation was performed on a reversed-phase column utilizing a gradient of water and acetonitrile, and data were acquired on a high-resolution MS in positive and negative ESI modes. Run order included solvent blanks and pooled QCs injected regularly; features with QC CV > 30% or present in blanks were removed. Raw data were processed for peak picking and alignment, annotated against public libraries, and normalized to internal standards and cell protein. Multivariate (PCA, OPLS-DA) and univariate tests with FDR correction identified differential features.

### Targeted lipidomics

Targeted lipidomics were performed by Sinotech Genomics (Shanghai, China). Lipid extraction used MTBE with class-matched isotopic internal standards added before extraction. Extracts were separated on a lipid-optimized reverse-phase column and analyzed by scheduled MRM on a triple-quadrupole MS. Transitions and collision energies were optimized with standards. Quantification used internal-standard normalization and calibration curves for absolute measurements; otherwise, semi-quantitative values were reported. QC samples and standard mixes were run periodically. Data were analyzed for species-level changes, class ratios (e.g., saturated/unsaturated TAG), and visualized by heatmap or clustering. Tests used included t-test or ANOVA with FDR correction.

### Co-immunoprecipitation (co-IP) and LC-MS

Non-denaturing IP buffer with protease and phosphatase inhibitors was used to lysate the Patu-8988 cells. The samples were then cleared and precleared with control IgG. Lysates were then incubated with anti-OAS1 or control anti-IgG, and anti-FASN or anti-IgG in another experiment, which were then captured on protein A/G agarose beads with rotation at uniform speed for 2 h at room temperature. Anti-FLAG M2 Magnetic Beads (M8823, Merck) were used in IP with OAS1 truncation and mutation. Samples with beads were then washed stringently, and a fraction of each group was reserved for Western blotting validation.

The rest of the samples were transported to OBiO Technology (Shanghai, China) for LC-MS. For proteomics, beads were reduced, alkylated, and digested with trypsin. Peptides were desalted and then detected by LC-MS as described above. Raw files were searched against UniProt with 1% FDR. Candidate interactors were defined by enrichment over IgG controls and validated by reciprocal Western blotting.

### Plasmid construction and site-directed mutagenesis

Plasmid construction and site-directed mutagenesis were delegated to OBiO Technology (Shanghai, China). The full-length human OAS1 (NM_002534.3) was cloned into the pCDNA3.1-3×Flag vector. To map the interaction domains, OAS1 truncation mutants, including N-terminal (NT, aa 1–104), Core (aa 105–345), and C-terminal (CT, aa 346–400), were generated by PCR amplification and inserted into the same vector. The catalytically inactive OAS1 mutant (D75/77A, Asp75 and Asp77 mutated to Ala) was generated using the QuikChange II Site-Directed Mutagenesis Kit (200523, Agilent) according to the manufacturer’s instructions. All constructs were verified by DNA sequencing. FASN was cloned into the pCDNA3.1-HA vector. Plasmids were transfected into cells using Lipofectamine 3000 (L3000001, Invitrogen) when the cell confluence reached 70%.

### Mouse models

Seven-week-old male nude mice (BALB/C-nu/nu) were used for the subcutaneous xenograft model (*n* = 5). Patu-8988 cells were injected subcutaneously in the inguinal regions of the mice at a concentration of 1 × 10^6^ cells/100 μL PBS. Tumor length was measured every 7 days after injection. The volume of the tumor was calculated as 4/3*π*(*L*/2)³. The endpoint was week 4 after injection. Tumors were harvested, measured, weighed, and processed for IHC staining.

Seven-week-old male C57BL/6 mice were used for the orthotopic transplantation model (*n* = 10). Luciferase-labeled KPC-1199 cells were injected into the pancreatic tail at a concentration of 1 × 10^5^ cells/100 μL PBS. Bioluminescence imaging was performed at 2 weeks using IVIS Spectrum through D-Luciferin, Potassium Salt D injection. Separate parallel cohorts were followed for survival endpoints. At the endpoint of week 4 after injection, the pancreas with tumors and the livers were collected. Tumor burden and metastases were recorded, and tissues were processed for IHC staining.

Mice were randomly assigned to experimental groups based on weight stratification to ensure balanced group distribution. No formal statistical methods were used to pre-determine sample size. The sample sizes were chosen based on previous experience with similar experimental models and established protocols in PDAC research, which have consistently demonstrated that the selected n values provide adequate power to detect biologically significant differences. Investigator blinding was not feasible during the administration of treatments in animal models. However, to ensure objective assessment, downstream histopathological analysis was performed by independent observers in a blinded manner. All animal experiments were performed in accordance with the Guide for the Care and Use of Laboratory Animals. The study protocol was reviewed and approved by the Institutional Animal Care and Use Committee (IACUC) of Fudan University (Approval No. 2025-HDYY-104).

### Statistical analysis

GraphPad Prism (v8.0), SPSS (v22.0), or R software (v4.0.2) were used in statistical analyses. ImageJ (v1.53c) was used in image analyses. In this study, all data are presented as mean ± standard deviation and tested two-sided. For datasets with *n* ≥ 5, the normality of distribution was assessed using the Shapiro–Wilk test, and homogeneity of variances was validated using the *F*-test or Levene’s test. For datasets with smaller sample sizes (*n* = 3), a normal distribution was assumed based on historical experimental consistency, and parametric tests were applied accordingly. For comparisons between two groups, a parametric Student’s *t*-test was performed. For data with more than two groups, one-way ANOVA followed by Tukey’s post hoc test was employed. Two-way ANOVA was used for time-course data analysis. Survival distributions were estimated using the Kaplan–Meier method and compared via the log-rank test, while Cox proportional hazards regression models were applied for multivariable survival analyses. For multi-omics datasets, *p*-values were adjusted for multiple testing using the Benjamini-Hochberg false discovery rate (FDR) correction. No data points were excluded from the analysis unless otherwise specified, and any exclusions were based on pre-established criteria such as technical failure. Statistical significance was considered as a *P* value < 0.05. Levels of significance have been annotated or symbolized in figures as follows: ns, *p* > 0.05; *p < 0.05; **p < 0.01; ***p < 0.001. In specific cases, an additional symbol (#) was used for specific group comparisons, as detailed in the respective figure legends.

## Results

### A screen of fatty acid metabolism-related genes highlights OAS1 from the OAS family

**Fig. 1:**
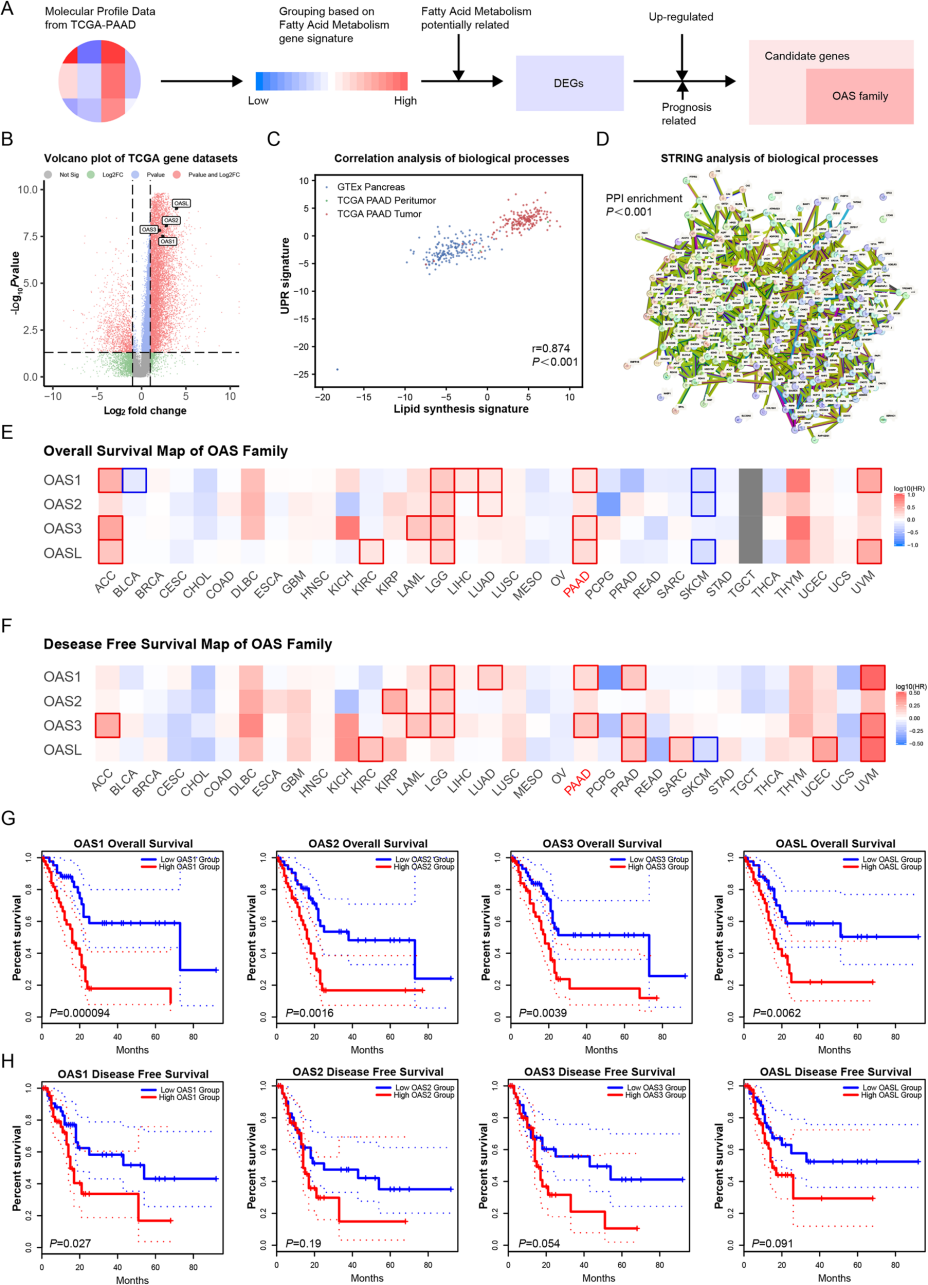
A screen of fatty acid metabolism-related genes highlights OAS1 from the OAS family. **A** Analysis workflow for fatty acid metabolism-based grouping in TCGA databases. **B** Expression of OAS1/2/3/L in FAM-high vs. FAM-low PAAD groups. **C** Correlation analysis of lipid synthesis signature and UPR signature in TCGA and GTEx databases. **D** Multiple proteins STRING analysis within homo sapiens. **E** Pan-cancer overall survival association map for OAS family members. **F** Pan-cancer recurrence-free-survival association map for OAS family members. **G** Quartile-based overall survival in the TCGA PAAD databases of each OAS gene. **H** Quartile-based disease-free survival in the TCGA PAAD databases of each OAS gene.

To identify genes associated with FAM in PDAC, we first stratified patients from the TCGA-PAAD cohort into two distinct clusters, FAM-high and FAM-low, based on the expression of a FAM hallmark gene signature (Fig. 1A). DEG analysis between these groups revealed a notable enrichment of the 2′-5′-Oligoadenylate Synthetase (OAS) gene family (Figs. 1B and S1A), a stratification whose stability was rigorously confirmed (Fig. S1B–D). Survival analysis indicated that patients in the FAM-high group exhibited significantly shorter OS and DFS compared to those in the FAM-low group (Fig. S1E, F). Furthermore, we observed a strong positive correlation between lipid synthesis and the UPR in TCGA-PAAD and GTEx pancreatic tissues (Fig. 1C), which was further corroborated by protein-level interaction networks through STRING analysis (Fig. 1D). These results support the possible involvement of OAS family members in the aggressive, high lipid metabolism phenotype of PDAC.

To further verify the detailed prognostic relevance of the OAS family, we first investigated the overall (Fig. 1E) and recurrence-free survival (Fig. 1F) of individual OAS family members across TCGA cancer types. Notably, within the PDAC cohort, OAS1 emerged as the only member whose high expression was significantly correlated with both poor OS and recurrence-free survival (RFS). Although other OAS members exhibited similar trends, their associations did not achieve statistical significance (Fig. 1G, H). Expression analysis across TCGA and GTEx databases further confirmed that while the entire OAS signature was generally up-regulated in tumor tissues, OAS1 demonstrated a particularly robust overexpression in PDAC (Fig. S1G–I). Collectively, these data establish the prognostic value of the OAS family members in cancer and specifically highlight OAS1 as a key driver associated with poor prognosis and lipid metabolic dysregulation in PDAC.

### OAS1 is up-regulated in PDAC and correlates with poor clinical outcome

**Fig. 2:**
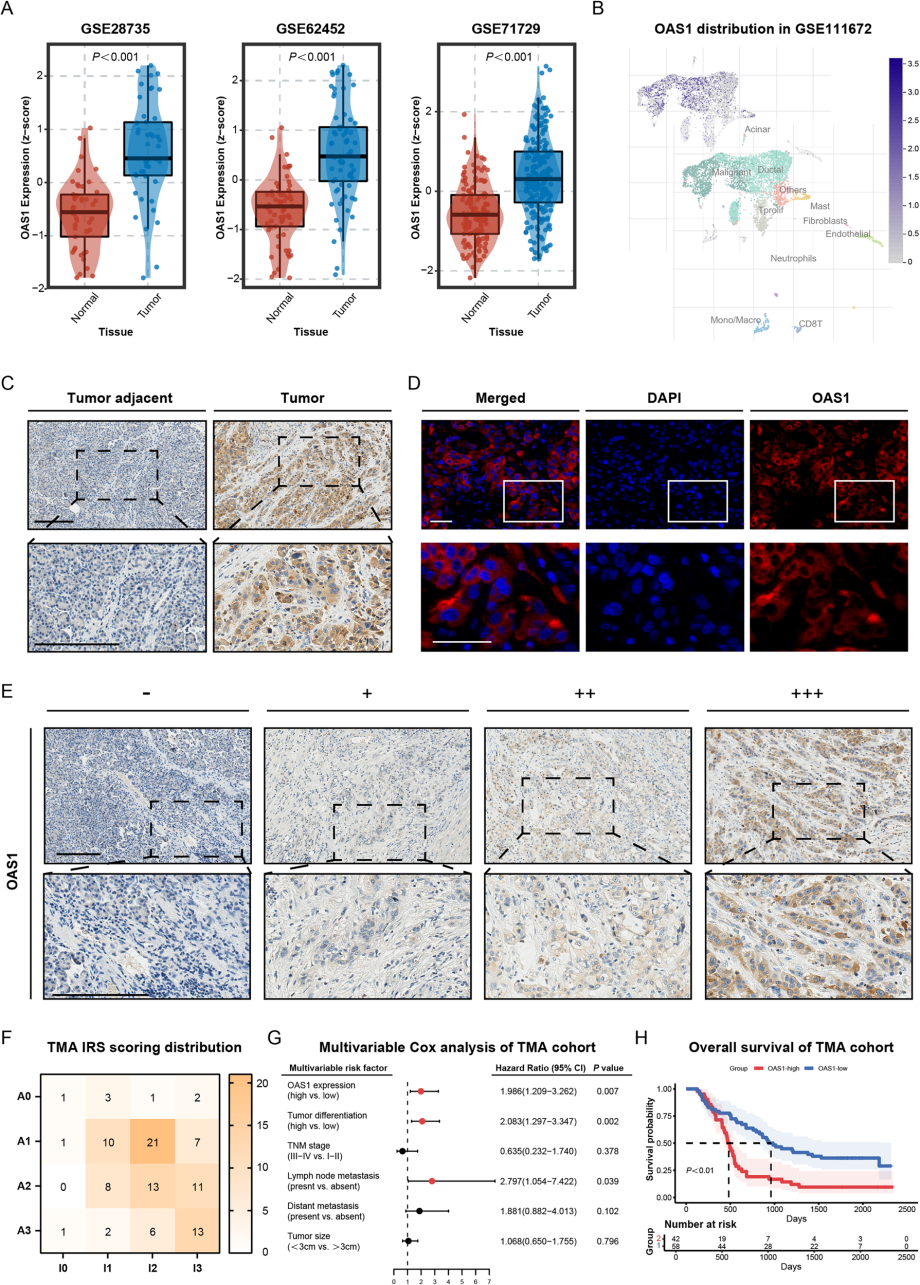
OAS1 expression is up-regulated in PDAC tissues and associates with poor outcome. **A** OAS1 expression by z-score between normal and tumor tissues in three databases. **B** Single-cell UMAP of PDAC colored by OAS1 expression by cell type in database GSE111672. **C** Representative IHC staining images of OAS1 in PDAC and adjacent tissue (scale bar: 200 μm). **D** Representative IF staining images of OAS1 in PDAC tissues with DAPI (scale bar: 50 μm). **E** Representative IHC staining images of OAS1 in PDAC TMA (scale bar: 200 μm). **F** PDAC TMA layout and case-level IRS heatmap for OAS1. **G** Multivariable Cox regression using OAS1 IRS (forest plot of HR and 95% CI). **H** Kaplan–Meier overall survival for OAS1 high vs. low groups in TMA by IRS. For IHC staining in (**C**, **E**) and IF staining in (**D**), primary antibodies were diluted as specified in Supplementary Table 1 (OAS1 1:200) and incubated overnight at 4 °C, followed by, respectively, corresponding secondary antibodies incubation for 1 h at room temperature. Visual signal was developed using DAB, and the sections were counterstained with hematoxylin for nuclear visualization.

We further explored the expression landscape of the OAS family in PDAC. Analysis of multiple public datasets, including GSE28735 [29, 30], GSE62452 [31], and GSE71729 [32], revealed that all four OAS members were significantly overexpressed in tumor tissues compared to normal pancreatic tissues (Figs. 2A and S2A–C). However, according to the results of single-cell sequencing, OAS1 was predominantly enriched within the malignant cell cluster, showing a distribution pattern notably stronger than that of its family counterparts in both the GSE11672 [33] (Figs. 2B and S2D, E) and CRA001160 cohorts [34] (Fig. S2F, G).

To validate these findings at the protein level, we evaluated OAS1 expression and localization in a clinical cohort from our own institution. Immunohistochemistry (IHC) staining revealed a marked elevation of OAS1 protein in PDAC tissues compared to paired adjacent non-tumor tissues (Fig. 2C). Furthermore, immunofluorescence (IF) staining demonstrated that OAS1 is primarily localized within the cytoplasm and perinuclear regions of PDAC cells (Fig. 2D).

To establish the clinical utility of OAS1, we performed IHC-based scoring on a TMA containing 100 PDAC samples. Patients were stratified into high and low OAS1 expression groups based on a composite score of staining intensity and area as IRS (Fig. 2E, F). Multivariable Cox regression analysis, adjusted for standard clinicopathological variables, identified high OAS1 expression as a robust independent prognostic factor for poor survival in our cohort (Fig. 2G). Consistent with the TCGA findings, patients in the OAS1-high group exhibited significantly shorter OS (Fig. 2H). These results confirm that OAS1 is up-regulated in malignant pancreatic cells and serves as an independent marker for predicting poor prognosis in PDAC patients.

### OAS1 promotes malignant phenotypes in PDAC cells independently of interferon signaling

**Fig. 3:**
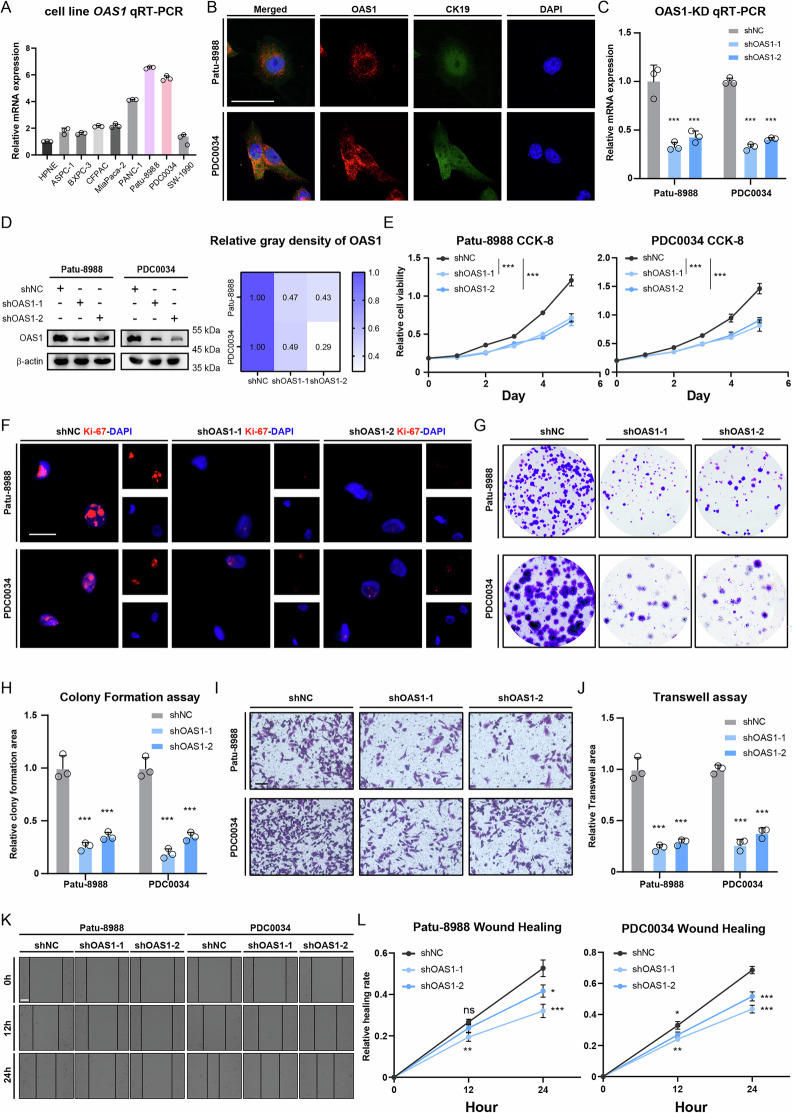
OAS1 promotes malignant phenotypes in PDAC cells in vitro. **A** Relative OAS1 mRNA expression levels in normal pancreatic ductal epithelial cells and PDAC cell lines by qRT-PCR. **B** IF staining for OAS1 and CK19 in Patu-8988 and PDC0034 with DAPI (scale bar: 50 μm). **C** qRT-PCR validation of OAS1 knockdown in Patu-8988 and PDC0034 (shNC vs. shOAS1). **D** Western blotting analysis for OAS1 knockdown with relative band gray densitometry heatmap. **E** CCK-8 growth curves of Patu-8988 and PDC0034 based on shNC vs. shOAS1. **F** IF staining for Ki-67 in Patu-8988 and PDC0034 with DAPI (scale bar: 50 μm). Colony-formation assay of OAS1 knockdown in Patu-8988 and PDC0034 (shNC vs. shOAS1) (**G**) with quantification (**H**). Transwell assay of OAS1 knockdown in Patu-8988 and PDC0034 (shNC vs. shOAS1) (**I**) with quantification (**J**) (scale bar: 200 μm). Wound-healing assay of OAS1 knockdown in Patu-8988 and PDC0034 (shNC vs. shOAS1) (**K**) with healing (closure) rates at indicated times (**L**) (scale bar: 200 μm). For IF staining in (**B**, **F**), primary antibodies were diluted as specified in Supplementary Table 1 (OAS1 1:200, Ki-67 1:1000) and incubated overnight at 4 °C, followed by Cy3-conjugated Goat anti-rabbit IgG (1:100) incubation for 1 h at room temperature. For WB assay in (**D**), primary antibodies (OAS1 1:1000, β-actin 1:1000) were diluted and incubated overnight at 4 °C followed by Goat Anti-Rabbit IgG H&L (HRP) (1:5000) incubation for 1 h at room temperature.

To evaluate the biological role of OAS1, we first profiled its baseline expression across various cell lines. Compared to HPNE cells, PDAC cell lines generally exhibited significantly elevated OAS1 mRNA levels (Fig. 3A). Based on their high endogenous expression, Patu-8988 and PDC0034 cells were selected for subsequent loss-of-function studies. Figure 3B displays the expression pattern of OAS1 by IF staining in these two PDAC cell lines.

While IFN-α treatment successfully induced ISG expression, which was effectively neutralized by an IFNAR2-blocking antibody (Fig. S3A, B) [35, 36], the pharmacological inhibition of the IFNAR2 axis failed to alter PDAC cell proliferation (Fig. S3C, D). These findings, coupled with the known immunosuppressed nature of the PDAC microenvironment [6], suggest that the pro-tumorigenic role of OAS1 in this context is likely uncoupled from canonical IFN signaling.

To evaluate the functional impact of OAS1 directly, we utilized shRNAs to achieve stable knockdown, which was validated at both the mRNA (Fig. 3C) and protein levels (Fig. 3D). OAS1 knockdown significantly impaired proliferation as measured by CCK-8 assays (Fig. 3E), Ki-67 staining (Fig. 3F), and colony formation assays (Fig. 3G, H) in both PDAC cell lines. Furthermore, OAS1 knockdown significantly reduced cell invasion in Transwell assays (Fig. 3I, J) and slowed cell migration in wound healing assays (Fig. 3K, L). Collectively, these results demonstrate that OAS1 is a critical driver of malignant phenotypes in PDAC cells in vitro.

### OAS1 knockdown inhibits lipid metabolism and ER stress in PDAC cells

**Fig. 4:**
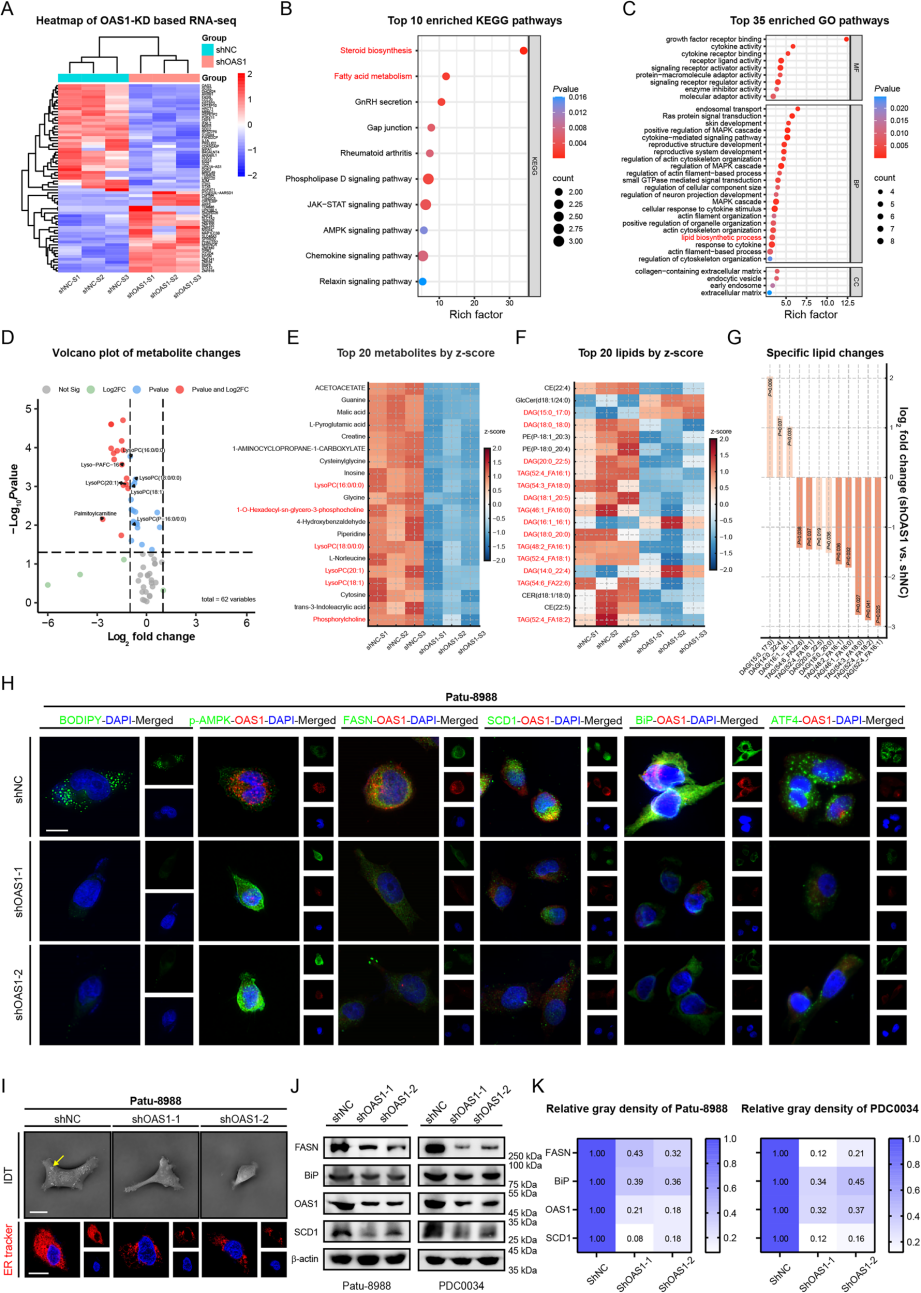
OAS1 knockdown inhibits lipid metabolism and ER stress in PDAC cells. **A** Differential gene heatmap of shOAS1 vs. shNC Patu-8988 by RNA-seq. **B** Top 10 enriched KEGG pathways of differentially expressed genes of shOAS1 vs. shNC Patu-8988 by RNA-seq. **C** Top 35 enriched GO pathways of differentially expressed genes of shOAS1 vs. shNC Patu-8988 by RNA-seq. **D** Differential metabolites comparing shOAS1 and shNC Patu-8988 by untargeted metabolomics. **E** Top 20 differential metabolites ranked by z-score according to untargeted metabolomics. **F** Top 20 differential lipids ranked by z-score according to targeted lipidomics. **G** Specific lipid changes between shOAS1 vs. shNC Patu-8988 by targeted lipidomics. **H** BODIPY lipid staining, and OAS1 and p-AMPK/FASN/SCD1/BiP/ATF4 IF staining images of Patu-8988 with DAPI (scale bar: 20 μm). **I** Live-cell imaging of lipid droplets and confocal ER Tracker staining images of Patu-8988 (Yellow arrow: lipid droplets; scale bar: 20 μm). Western blotting of lipid- and ER-related proteins (**J**) with gray densitometry heatmap (**K**). For IF staining in (**H**), primary antibodies were diluted as specified in Supplementary Table 1 (OAS1 1:200, FASN 1:100, p-AMPK 1:200, SCD1 1:100, BiP 1:100, ATF4 1:200) and incubated overnight at 4 °C, followed by either FITC-conjugated Goat anti-rabbit IgG (1:100) or Cy3-conjugated Goat anti-rabbit IgG (1:100) incubation for 1 h at room temperature. BODIPY (**H**) and ER tracker (**I**) were incubated at room temperature for 20 min. For WB assay in (**J**), primary antibodies (OAS1 1:1000, FASN 1:5000, BiP 1:1000, SCD1 1:2000, β-actin 1:1000) were diluted and incubated overnight at 4 °C, followed by Goat Anti-Rabbit IgG H&L (HRP) (1:5000) incubation for 1 h at room temperature.

To gain molecular insight into the pathways regulated by OAS1 in PDAC, we performed RNA-seq on shOAS1 and control Patu-8988 cells. Differential expression analysis revealed distinct transcriptomic signatures (Fig. 4A), with KEGG and GO analyses identifying lipid metabolism as the most significantly enriched pathway (Fig. 4B, C). Subsequent untargeted metabolomics confirmed broad shifts in metabolite profiles, particularly within the lipidome (Figs. 4D, E and S4A, B). To achieve higher molecular resolution, we conducted targeted lipidomics, which revealed that OAS1 knockdown significantly decreased the levels of multiple saturated triacylglycerol (TAG) species while increasing several unsaturated diacylglycerol (DAG) species (Fig. 4F, G). This specific lipid remodeling is a hallmark of reduced de novo lipogenesis, a process heavily dependent on Fatty Acid Synthase (FASN) [37–40].

Reinforcing the non-canonical nature of this axis, IFNAR2 neutralization failed to affect FASN-mediated metabolic output (Fig. S3E–G), further confirming that the OAS1-driven lipid phenotype is uncoupled from IFN signaling. Given that the accumulation of saturated lipids is a known trigger for the UPR [41–43], we investigated which UPR branch was primarily engaged. Through pharmacological inhibition and downstream target analysis, we determined that the PERK-eIF2α-ATF4 signaling axis was the primary pathway activated by OAS1, whereas the IRE1 and ATF6 arms remained relatively less affected (Fig. S4C–F).

To validate these findings, we assessed lipid metabolism indicators in parallel with OAS1 levels. Immunofluorescence (IF) staining demonstrated that OAS1 knockdown concurrently reduced BODIPY-stained lipid droplets, lowered FASN and SCD1 protein levels, and diminished markers of ER stress, including BiP expression and nuclear ATF4 localization (Figs. 4H and S4G). Live-cell imaging using BODIPY and ER-Tracker further visualized the real-time reduction of lipid droplets and ER expansion following OAS1 depletion (Figs. 4I and S4H). These phenotypic changes were further validated at both the mRNA (Fig. S4I) and protein levels (Fig. 4J, K). Together, these data indicate that OAS1 drives a FASN-related lipogenic state characterized by saturated lipid accumulation, ER stress, and the activation of the adaptive PERK-ATF4 ER stress response in PDAC cells.

### OAS1 interacts with FASN by the Core domain to drive lipid accumulation in PDAC cells

**Fig. 5:**
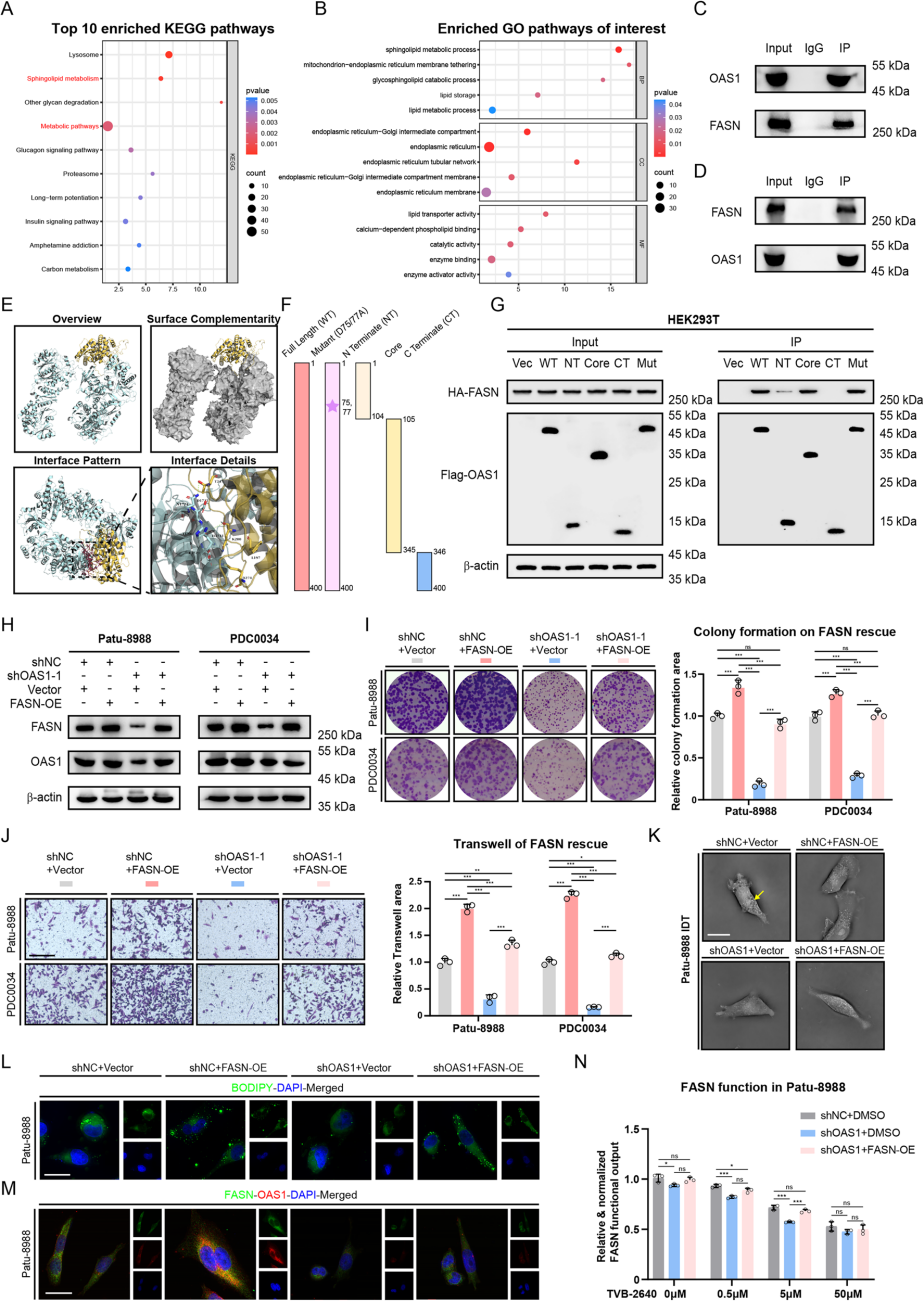
OAS1 interacts with FASN by the Core domain and increases lipid load in PDAC cells. **A** Top 10 enriched KEGG pathways of the pulldown proteins by co-IP of OAS1 and LC-MS. **B** Enriched GO pathways of interest of the pulldown proteins by co-IP of OAS1 and LC-MS. Western blotting validation of OAS1-FASN by co-IP of OAS1 (**C**) and co-IP of FASN (**D**). **E** Molecular docking models illustrating the interaction between OAS1 and FASN, including an overview of the predicted complex structure, surface complementarity between the OAS1-FASN interface, interface patterns, and detailed views of key interfacial residues and hydrogen bonding. **F** Schematic representation of OAS1 Full Length (WT), catalytically inactive mutant (D75/77A), and truncation mutants including N-terminal (NT, aa 1–104), Core (aa 105–345), and C-terminal (CT, aa 346–400). **G** Co-IP assay in HEK293T cells co-transfected with HA-tagged FASN and Flag-tagged OAS1 variants (WT, NT, Core, CT, or Mut) with β-actin as the loading control for Input. **H** Western blotting of FASN and OAS1 expression across indicated groups of Patu-8988 and PDC0034. **I** Colony-formation assay across indicated groups of FASN-rescue of Patu-8988 and PDC0034. **J** Transwell assay across indicated groups of FASN-rescue of Patu-8988 and PDC0034 with quantification (scale bar: 200 μm). **K** Live-cell lipid-droplet imaging across indicated groups of FASN-rescue of Patu-8988 (Yellow arrow: lipid droplets; scale bar: 20 μm). **L**, **M** BODIPY lipid staining (**M**), and OAS1 and FASN IF staining images of Patu-8988 with DAPI (indicated groups of FASN-rescue) (scale bar: 20 μm). **N** Relative FASN function across indicated groups of FASN-rescue Patu-8988 with TVB-2640 treatment. BODIPY (**L**) was incubated at room temperature for 20 min. For IF staining in (**M**), primary antibodies were diluted as specified in Supplementary Table 1 (OAS1 1:200, FASN 1:100) and incubated overnight at 4 °C, followed by either FITC-conjugated Goat anti-rabbit IgG (1:100) or Cy3-conjugated Goat anti-rabbit IgG (1:100) incubation for 1 h at room temperature. OAS, FASN, and IgG primary antibodies for IP were diluted at 1:100 (**C**, **D**). Anti-Flag M2 affinity beads were used in (**G**). For WB assay in (**G**, **H**), primary antibodies (OAS1 1:1000, FASN 1:5000, Flag 1:5000, HA 1:5000, β-actin 1:1000) were diluted and incubated overnight at 4 °C, followed by Goat Anti-Rabbit IgG H&L (HRP) (1:5000) incubation for 1 h at room temperature.

To further identify the interacting partners with whom up-regulated OAS1 promotes lipid metabolism in PDAC, the endogenous protein was immunoprecipitated and analyzed by liquid chromatography-mass spectrometry. Exhilaratingly, the pulldown list was significantly enriched for lipid- and ER-related terms by KEGG and GO analysis, and FASN was pointed out among the prominent candidate interactors (Fig. 5A, B). Western blotting analysis of forward and reciprocal immunoprecipitations with OAS1 and FASN confirmed the interaction (Fig. 5C, D).

Next, to gain structural insights into the OAS1-FASN interaction, we performed molecular docking using the HDOCK server [44–54]. The top-ranked model (Confidence Score: 0.9365) predicted a strong interface between the Core domain of OAS1 and FASN, involving key residues such as 1725(FASN)-247(OAS1) as visualized by PyMOL in Fig. 5E (Supplementary Table 4). To experimentally map the interaction domains, we constructed a series of Flag-tagged OAS1 truncations and a catalytically inactive mutant (D75/77A, Mut) (Fig. 5F) [55–58]. Co-IP results in HEK293T cells revealed that while the full-length (WT) and Mut proteins strongly interacted with FASN, the Core domain (aa 105–345) was the minimal region required for this binding (Fig. 5G). In contrast, the C-terminal (CT) truncation failed to bind FASN.

Next, functional rescue experiments were conducted in shOAS1-transfected Patu-8988 and PDC0034 cells. Re-expression of WT, the Core domain, or the D75/77A mutant significantly restored cell viability, colony formation, and migration (Fig. S5A–C), as well as FASN functional output (Fig. S5D) and lipid droplet accumulation (Fig. S5E). Notably, the enzymatic activity-deficient mutant (Mut) performed similarly to the WT in all assays, whereas the CT truncation showed no rescue effect. Collectively, these results provide evidence that the OAS1-FASN axis promotes PDAC progression through the OAS1 Core domain-mediated protein-protein interaction, independent of OAS1’s canonical 2′-5′ oligoadenylate synthetase activity.

To confirm the lipogenic capacity of FASN in PDAC cells, we overexpressed FASN in wild-type PDAC cells and observed a corresponding increase in lipid droplet accumulation (Fig. S6A–C). However, considering the lack of prognostic significance of FASN expression alone in PDAC cohorts (Fig. S6D–F), we hypothesized that FASN’s pro-tumorigenic effect in PDAC might depend on specific regulatory partners, such as OAS1. To test this hypothesis, we performed functional rescue experiments by overexpressing FASN in OAS1-deficient Patu-8988 and PDC0034 cells. qRT-PCR (Fig. S6G) and western blot analysis confirmed the successful restoration of FASN levels in shOAS1 cells (Figs. 5H and S6H). Functionally, FASN overexpression significantly rescued the impairments in cell proliferation (Fig. S6I) and colony formation capacity (Fig. 5I) induced by OAS1 knockdown. Similarly, the reduced migratory potential of shOAS1 cells was effectively reversed upon FASN restoration (Figs. 5J and S6J). And as expected, re-expressing FASN in OAS1-knockdown cells was sufficient to restore key molecular markers of lipid synthesis and ER stress, and this rescue was visualized by the restoration of lipid droplets and the colocalization of OAS1 and FASN (Figs. 5K–M and S6K–N).

Furthermore, we evaluated FASN function across a concentration gradient of the FASN inhibitor TVB-2640. In Patu-8988 cells, higher OAS1 levels were associated with stronger functional persistence, even when normalized to the protein expression levels of each group (Fig. 5N), which indicates that OAS1 maintains the function of FASN in PDAC cells. Taken together, these results demonstrate that rather than relying on its canonical enzymatic activity, OAS1 functions as a vital architectural partner for FASN, providing the structural support necessary to sustain FASN-driven lipid accumulation and tumor growth in PDAC.

### OAS1-FASN-driven lipid overload engages an ATF4-mediated adaptive ER stress response in PDAC cells

**Fig. 6:**
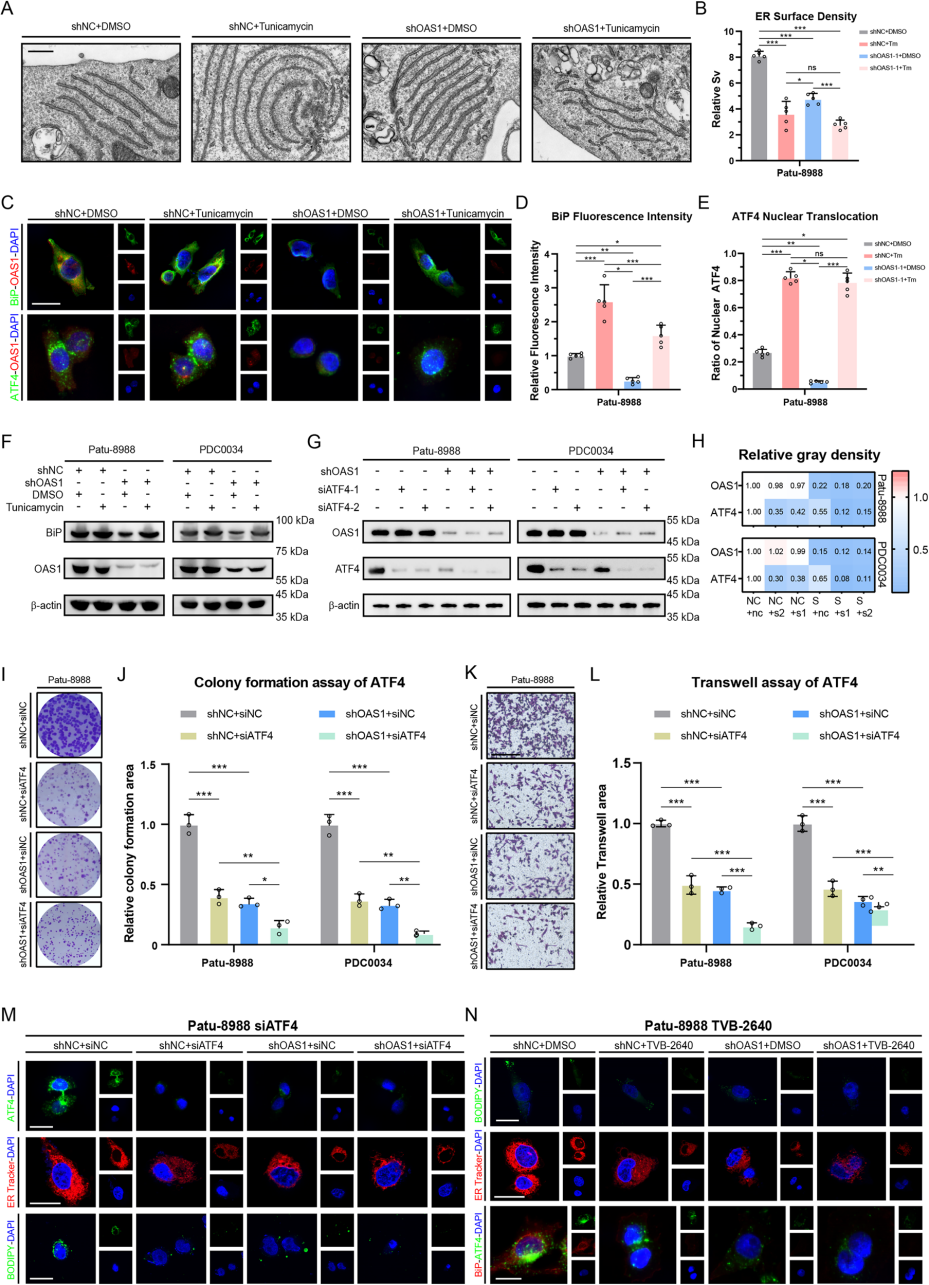
OAS1-FASN-driven lipid overload engages an ATF4-mediated adaptive ER stress response in PDAC cells. Transmission electron microscopy images of ER ultrastructure (**A**) and statistical analysis of ER surface density (**B**) in indicated groups of Patu-8988 based on OAS1 knockdown and Tunicamycin treatment (scale bar: 500 nm). BODIPY lipid staining and BiP-ATF4 IF staining images (**C**), statistical analysis of BiP intensity (**D**), and ATF4 nuclear translocation ratio (**E**) of indicated groups of Patu-8988 based on OAS1 knockdown and Tunicamycin treatment with DAPI (scale bar: 20 μm). **F** Western blotting of BiP and OAS1 expression across indicated groups of Patu-8988 and PDC0034. Western blotting analysis (**G**) and corresponding gray density heatmaps (**H**) showing the protein levels of OAS1 and ATF4 in the indicated groups. Representative images (**I**) and statistical analysis (**J**) of colony formation assay of Patu-8988 based on ATF4 knockdown upon OAS1 deficiency. Representative images (**K**) and statistical analysis (**L**) of the Transwell assay of Patu-8988 and PDC0034 based on ATF4 knockdown upon OAS1 deficiency. **M** Immunofluorescence staining of ATF4, ER-Tracker, and BODIPY in Patu-8988 cells across the four experimental groups (scale bar: 20 μm). **N** BODIPY lipid staining, ER Tracker staining, and BiP-ATF4 IF staining images of indicated groups of Patu-8988 based on OAS1 knockdown and TVB-2640 treatment with DAPI (scale bar: 20 μm). For IF staining in (**C**, **M**, **N**), primary antibodies were diluted as specified in Supplementary Table 1 (OAS1 1:200, BiP 1:100, ATF4 1:200) and incubated overnight at 4 °C, followed by either FITC-conjugated Goat anti-rabbit IgG (1:100) or Cy3-conjugated Goat anti-rabbit IgG (1:100) incubation for 1 h at room temperature. BODIPY and ER tracker (**M**, **N**) were incubated at room temperature for 20 min. For WB assay in (**F**, **G**), primary antibodies (OAS1 1:1000, BiP 1:1000, ATF4 1:1000, β-actin 1:1000) were diluted and incubated overnight at 4 °C, followed by Goat Anti-Rabbit IgG H&L (HRP) (1:5000) incubation for 1 h at room temperature.

To further characterize how OAS1 influences ER homeostasis, we first treated OAS1-knockdown cells with Tunicamycin (Tm) to exogenously induce ER stress [59]. Transmission electron microscopy (TEM) imaging confirmed that Tm treatment induced a typically structured ER morphology characteristic of high stress, consistent with OAS1 expression change and representational lipid accumulation [37, 42], which revealed that high OAS1 expression is associated with a robust ER network, whereas OAS1 knockdown significantly reduced ER surface density and lipid load (Figs. 6A, B and S7A–C). Furthermore, while Tm treatment was used to challenge the cells, OAS1-deficient cells showed a significantly blunted stress response compared to control cells, characterized by lower BiP fluorescence intensity and expression, and a reduced ratio of ATF4 nuclear translocation (Figs. 6C–F and S7D, E). These findings suggest that the OAS1-driven lipid metabolism is inherently linked to a high-capacity, adaptive ER system.

Next, we performed siRNA-mediated knockdown of ATF4 in Patu-8988 and PDC0034 cells. qRT-PCR and Western blotting confirmed the successful reduction of ATF4 expression (Figs. 6G, H and S7F, G). Functional assays showed that ATF4 knockdown significantly suppressed cell viability and colony formation, mimicking the effects of OAS1 deficiency (Figs. 6I, J and S7H, I). Notably, in OAS1-deficient cells, the additional knockdown of ATF4 led to a further reduction in malignant potential, including decreased proliferation (Figs. 6I, J and S7H, I) and invasion (Figs. 6K, L and S7J). Immunofluorescence staining further confirmed that loss of ATF4 significantly decreased the accumulation of lipid droplets and altered ER-Tracker distribution (Figs. 6M and S8A), indicating a collapse of the metabolic and structural support provided by the OAS1-FASN-ATF4 axis. These results demonstrate that ATF4 is a key functional mediator required to sustain the tumorigenic growth under the metabolic stress driven by OAS1.

To explore the functional dependency of this metabolic axis, we tested whether the OAS1-driven state creates a metabolic susceptibility to pharmacological FASN inhibition using TVB-2640, a potent FASN inhibitor. Dose-response assays revealed that OAS1-deficient cells were significantly more sensitive to TVB-2640 than control cells, with IC_50_ values dropping from 0.697 to 0.149 μM in Patu-8988 (Fig. S8B) and from 2.075 to 0.399 μM in PDC0034 cells (Fig. S8C). Consistent with the IC_50_ shifts, functional assays showed that combining OAS1 knockdown with TVB-2640 treatment resulted in a more profound suppression of colony formation (Fig. S8D) and cell invasion (Fig. S8E) compared to single-agent treatments, indicating a clear additive effect in suppressing PDAC malignancy. Immunofluorescence staining confirmed that TVB-2640 treatment led to a significant depletion of lipid droplets and altered the ER network (Figs. 6N and S8F). Importantly, similar to the effects of OAS1 knockdown, TVB-2640 restricted the nuclear translocation of ATF4 and reduced BiP expression (Figs. 6N and S8F), suggesting that the pharmacological blockade of the OAS1-FASN axis prevents cells from raising an adaptive ER stress response.

Collectively, these data demonstrate that the OAS1-FASN-ATF4-mediated ER stress axis is a critical functional dependency in PDAC lipid metabolism, where OAS1 serves as a key regulatory metabolic modifier.

### OAS1-FASN axis promotes PDAC progression and lipid metabolism in vivo

**Fig. 7:**
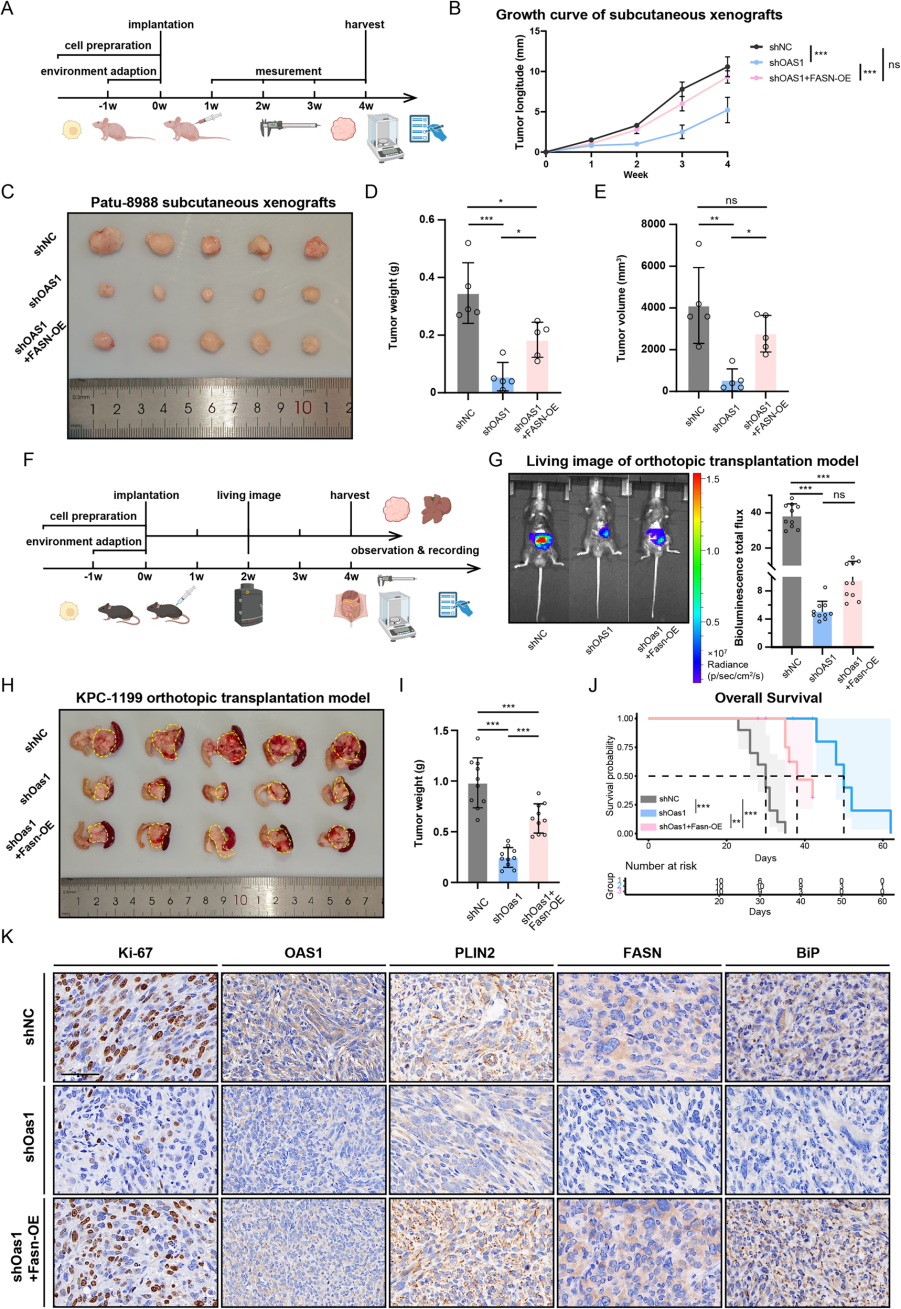
OAS1-FASN axis promotes PDAC progression and lipid metabolism in vivo. **A** Workflow diagram for the subcutaneous xenograft using stably transfected Patu-8988 cells. **B** Tumor growth curves by longitude over time of the three groups (*n* = 5 for each group). **C** Gross tumor images of Patu-8988 subcutaneous xenografts at endpoint with scale reference (*n* = 5 for each group). Statistical analysis of the weight (**D**) and volume (**E**) of the subcutaneous xenografts of the three groups (*n* = 5 for each group). **F** Workflow diagram for the KPC-1199 orthotopic transplantation model. **G** Representative living imaging of three groups of mouse models and quantification analysis (*n* = 10 for each group). **H** Gross tumor images of KPC-1199 orthotopic transplantation tumors at endpoint with scale reference (*n* = 10 for each group). **I** Statistical analysis of the weight of the orthotopic transplantation tumors of the three groups (*n* = 10 for each group). **J** Kaplan–Meier survival curves for parallel cohorts (*n* = 10 for each group). **K** Representative IHC staining for Ki-67, OAS1, PLIN2, FASN, and BiP of KPC-1199 orthotopic transplantation tumors (scale bar: 200μm). For IHC staining in (**K**), primary antibodies were diluted as specified in Supplementary Table 1 (Ki-67 1:1000, OAS1 1:200, PLIN2 1:200, FASN 1:1000, BiP 1:100) and incubated overnight at 4 °C, followed by HRP-conjugated secondary antibodies incubation for 1 h at room temperature. Visual signal was developed using DAB, and the sections were counterstained with hematoxylin for nuclear visualization.

To evaluate whether the OAS1-FASN axis promotes PDAC progression in vivo, we used OAS1-deficient Patu-8988 with or without FASN overexpression to establish subcutaneous xenograft models, as shown in Fig. 7A (*n* = 5). Consistent with our in vitro data, OAS1 knockdown significantly suppressed tumor growth, as measured by tumor volume and endpoint weight. Importantly, while overexpression of FASN in these OAS1-knockdown cells partially rescued tumor growth, the stable overexpression of FASN significantly rescued this phenotype (Fig. 7B–E). Specifically, FASN overexpression led to a marked restoration of tumor weight (*P* = 0.0492) and tumor volume (*P* = 0.0327) compared to the shOAS1 group. IHC analysis of tumor sections confirmed that tumors from the shOAS1 group had lower levels of the proliferation marker Ki-67, the lipid marker PLIN2, and the ER stress marker BiP, while FASN overexpression restored their expression (Fig. S9A).

Similarly, in the orthotopic transplantation model (*n* = 10) (Fig. 7F), although the suppression of tumor growth induced by OAS1 deficiency was not significantly attenuated by FASN restoration as mid-term living image showed (*P* = 0.0749) (Fig. 7G), end-point tumor burden (Fig. 7H) and tumor weight measurements (Fig. 7I) confirmed that FASN-OE partially reversed the growth inhibition seen in OAS1-deficient tumors (*P* = 0.0001 for tumor weight). Furthermore, survival analysis revealed that the extended lifespan observed in the shOAS1 group was significantly shortened upon FASN overexpression (*P* = 0.0024) (Fig. 7J), indicating that the OAS1-FASN axis is a critical driver of PDAC malignancy and mortality. IHC analysis of the orthotopic tumors confirmed the same changes in Ki-67, PLIN2, and BiP expression observed in the subcutaneous model (Fig. 7K).

**Fig. 8:**
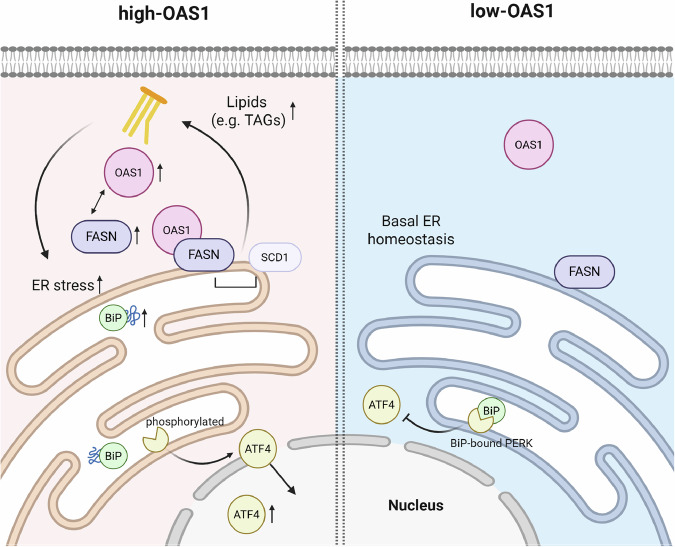
Model of the OAS1-FASN-ER stress axis in PDAC. This model summarizes and shows that OAS1 interacts with FASN. Lipid (e.g., triglyceride) synthesis rises, creating a higher lipid load with endoplasmic reticulum stress and activates the adaptive PERK-ATF4 pathway, with up-regulated BiP and nuclear ATF4. This whole process creates a high-lipid metabolic state and ultimately supports cell malignant behavior and tumor progression, while cells with lower OAS1 show a relatively metabolically steady state.

Furthermore, preliminary observation of liver metastases showed that metastatic burden was consistent with the primary tumor phenotypes. Qualitatively, OAS1 knockdown led to fewer detectable metastatic foci, while FASN overexpression was associated with an increased incidence of liver metastasis (*n* = 7) (Fig. S9B, C). Taken together, these in vivo studies demonstrate that OAS1 is a potent driver of PDAC tumor growth and suggest its contribution to metastatic potential, functioning primarily by supporting FASN-driven lipid metabolism (Fig. 8).

## Discussion

PDAC is defined by its lethality and a unique tumor microenvironment that lacks nutrients and oxygen [60]. To survive and proliferate in these conditions, PDAC cells undergo extensive metabolic reprogramming [61–63]. Dysregulated lipid metabolism is a key feature of this adaptation, providing substrates for membrane synthesis, energy storage, and intercellular signaling. Emerging evidence highlights that such metabolic plasticity is intrinsically linked to the hallmark chemoresistance of PDAC, where lipid-related genes often intersect with oncogenic pathways to increase therapeutic stress. This metabolic-signaling crosstalk not only fuels tumor aggressiveness but also offers a reservoir of potential biomarkers for predicting prognosis and drug sensitivity [64, 65]. While many studies have described the connection between altered lipid metabolism and PDAC progression, the specific molecular regulators participating in these processes remain incompletely understood [66]. Identifying novel axes, such as the OAS1-FASN signaling described here, is therefore crucial for uncovering possible new therapeutic vulnerabilities to overcome treatment resistance.

Among the various lipid metabolic processes, FAM and the synthesis of triacylglycerols (TAGs) are of great importance in PDAC. TAGs play significant roles in lipid storage and can buffer excess fatty acids, whose accumulation also reflects a hyper-lipogenic state [67, 68]. In this study, we screened the TCGA-PAAD database using a FAM signature and identified OAS1 as a prominent regulator of the PDAC lipidome, a finding we validated through multi-omics and targeted lipidomics. Our analysis of datasets and clinical samples confirms that OAS1 expression is significantly up-regulated in tumor tissues and independently correlates with poor patient outcomes. At the cellular level, OAS1 promotes cell proliferation, migration, and invasion, while in vivo models demonstrate that its depletion significantly reduces tumor burden. Analysis of single-cell sequencing datasets further refines this observation, revealing that OAS1 is predominantly distributed within malignant epithelial cells, showing higher specificity than other family members. However, it is important to acknowledge that the PDAC TME, characterized by dense desmoplasia and immune infiltration [4, 6, 60], likely provides a metabolic and signaling context that further modulates OAS1 activity. While our data highlight the intrinsic role of OAS1 in tumor cells, future studies should explore how the stromal and immune compartments interact with this epithelial metabolic axis.

OAS1 and the members of the OAS family are classically recognized as ISGs with established antiviral roles. Recent reports, however, suggest their broader functions in oncology, including immune regulation and cell proliferation. For instance, OASL was recently shown to reprogram lipid metabolism to promote tumor growth through enhancing mRNA translation [28], and OAS1 itself has been noted as a prognostic marker in several cancers [23–27]. Notably, a central finding of our study is that the pro-tumorigenic function of OAS1 in PDAC is uncoupled from its canonical interferon-dependent enzymatic activity. Our data using truncation mapping and the enzymatically inactive D75/77A mutant provide evidence that OAS1 operates as a structural scaffold rather than a catalytic enzyme in this context. This non-canonical role is further supported by our molecular docking analysis, which suggests that OAS1 interacts with the Ketoacyl Synthase (KS) and Enoylreductase (ER) domains of FASN (residues 42-49 and 1694-1784, respectively). Given that FASN functions as a head-to-head homodimer [38, 69, 70], we propose a model in which OAS1 acts as a molecular partner for the FASN dimer. By binding to these specific interfaces, OAS1 may stabilize the active conformation of the FASN complex or facilitate the efficient channeling of substrates between active sites, thereby maximizing the functional output of FASN. These findings highlight OAS1 as a structural regulator that repositions this classical antiviral protein into a central metabolic pathway. Such an IFN-independent role is consistent with the profoundly immunosuppressed nature of the PDAC microenvironment, where canonical immune-related signaling is frequently attenuated [71, 72].

The consequence of this OAS1-FASN interaction is a remodeling of the cellular lipid metabolic flux, characterized by the enrichment of saturated lipids. We hypothesize that this shift in lipid composition provides the potential link to the ATF4-mediated adaptive ER stress response [16]. The accumulation of saturated lipids is a known factor that alters the biophysical properties of the ER membrane [41, 42], which may in turn activate the PERK-eIF2α-ATF4 signaling axis [17, 20, 43]. Our data suggest that in PDAC cells, this lipid-driven ER stress functions as an adaptive response, thereby supporting cell survival under high metabolic demands. In this model, the OAS1-FASN axis appears to bridge de novo lipogenesis with an ER stress response that facilitates tumor progression. Despite these findings, several limitations of the present study should be noted. Firstly, our pharmacological assessment using TVB-2640 was conducted in cell culture systems. Secondly, the liver metastasis results remain preliminary, where these observations were descriptive and lacked the statistical power of dedicated experimental models, such as spontaneous tumor models, which are required to formally evaluate metastatic potential. Importantly, cell-free biochemical assays will be required to define the exact impact of OAS1 on FASN activity. Further validation of this metabolic circuit in more complex in vivo systems will be essential to fully characterize its biological and translational relevance in PDAC.

In summary, our study reveals a metabolism-related mechanism by which OAS1 promotes PDAC progression via a structural interaction with FASN. By driving the accumulation of saturated lipids and triggering an adaptive ER stress response, the OAS1-FASN axis defines a regulatory metabolic state in pancreatic cancer. Our work establishes OAS1 as a non-canonical metabolic regulator and a potent prognostic biomarker, suggesting that modulating this lipid-ER stress axis represents a potential avenue for exploring the metabolic vulnerabilities of PDAC.

## Conclusion

Our study identifies OAS1 as the key member of the OAS family with clear prognostic and functional relevance in PDAC. We demonstrate that OAS1 promotes the malignant phenotypes of PDAC cells by reprogramming cellular metabolism. Mechanistically, OAS1 interacts directly with FASN, maintaining its functional protein levels to drive a high-lipid metabolic state with an ATF4-mediated adaptive ER stress response. These findings elucidate a functional role of OAS1 in PDAC metabolism and tumor progression, suggesting the OAS1-FASN axis as a potential biomarker and a candidate pathway for further metabolic investigation in PDAC.

## Supplementary information

Tables

Supplementary figure legends

Fig. S1

Fig. S2

Fig. S3

Fig. S4

Fig. S5

Fig. S6

Fig. S7

Fig. S8

Fig. S9

Original western blots

## Data Availability

The data are available within the “Methods and materials,” Supplementary Information, or available from the authors upon reasonable request.
