# Supplementary material for: The OAS1-FASN axis promotes pancreatic cancer by coordinating lipogenic stress and the unfolded protein response
Source: Cell Death Dis. 2026 Jun 3;17(1):696. doi: 10.1038/s41419-026-08922-8 (PMC13448175; doi:10.1038/s41419-026-08922-8)
Supplement: Supplementary file 1 [file 41419_2026_8922_MOESM1_ESM.docx]

**Supplementary Table 1. Antibodies and dilution ratio in various assays.**

| **Target protein** | **Dilution ratio** | **Cat. No. and manufacturer** |
| --- | --- | --- |
| OAS1 | 1:200 (IHC, IF); 1:1000 (WB); 1:100 (IP) | 82883-1-RR, Proteintech |
| FASN | 1:1000 (IHC); 1:100 (IF); 1:5000 (WB); 1:100 (IP) | 10624-2-AP, Proteintech |
| p-AMPK | 1:200 (IF) | 83924-1-RR, Proteintech |
| Ki-67 | 1:1000 (IHC, IF) | ab15580, Abcam |
| SCD1 | 1:100 (IF); 1:2000 (WB) | 28678-1-AP, Proteintech |
| GRP78/BiP | 1:100 (IHC, IF); 1:1000 (WB) | 11587-1-AP, Proteintech |
| ATF4 | 1:200 (IF); 1:1000 (WB) | 10835-1-AP, Proteintech |
| PLIN2 | 1:200 (IHC); | 15294-1-AP, Proteintech |
| N-cadherin | 1:2000 (IHC); | 22018-1-AP, Proteintech |
| β-actin | 1:1000 (WB) | ab8227, Abcam |
| IgG | 1:100 (IP) | 30000-0-AP, Proteintech |
| Flag tag | 1:5000 (WB) | 66008-4-Ig, Proteintech |
| HA tag | 1:5000 (WB) | 51064-2-AP, Proteintech |
| Goat Anti-Rabbit IgG H&L (HRP) | 1:2000 (IHC); 1:5000 (WB) | ab205718, Abcam |
| FITC-conjugated Goat anti-rabbit IgG | 1:100 | GB22303, Servicebio |
| Cy3-conjugated Goat anti-rabbit IgG | 1:100 | GB21303, Servicebio |

**Supplementary Table 2. Sequences or transcripts in gene knockdown and overexpression assays.**

| **Name** | **Sequence 5’ to 3’ / Transcripts** |
| --- | --- |
| shOAS1-1 (human) | CCATGCTTTTAAGAATTGGGAT |
| shOAS1-2 (human) | CCAAATCTATGTCAAGCTCAT |
| shATF4-1 (human) | GCCAAGCACUCAAACUCAU |
| shATF4-2 (human) | GAGCAUUCCUULAGUGAUA |
| shOas1 (mouse) | CATGAGTTGCTCACTGTCTTT |
| OAS1 (Full length, human) | NM_002534.3 |
| FASN (human) | NM_004104.5 |
| Fasn (mouse) | NM_007988.3 |

**Supplementary Table 3. Primer sequences used in qRT-PCR assays.**

| **Target gene** | **Forward sequence** | **Reverse sequence** |
| --- | --- | --- |
| *OAS1* | TGTCCAAGGTGGTAAAGGGTG | CCGGCGATTTAACTGATCCTG |
| *OAS1*(CT) | TCCAACTCTGCAGATGATGAG | GGTCCAGTCCTCCTCTGCCT |
| *FASN* | AAGGACCTGTCTAGGTTTGATGC | TGGCTTCATAGGTGACTTCCA |
| *SCD1* | TCTAGCTCCTATACCACCACCA | TCGTCTCCAACTTATCTCCTCC |
| *BiP* | CATCACGCCGTCCTATGTCG | CGTCAAAGACCGTGTTCTCG |
| *ATF4* | ATGACCGAAATGAGCTTCCTG | GCTGGAGAACCCATGAGGT |
| *ISG15* | CGCAGATCACCCAGAAGATCG | TTCGTCGCATTTGTCCACCA |
| *IFIT1* | TTGATGACGATGAAATGCCTGA | CAGGTCACCAGACTCCTCAC |
| *MX1* | GTTTCCGAAGTGGACATCGCA | CTGCACAGGTTGTTCTCAGC |
| *CXCL10* | GTGGCATTCAAGGAGTACCTC | TGATGGCCTTCGATTCTGGATT |
| *DDIT3* | GGAAACAGAGTGGTCATTCCC | CTGCTTGAGCCGTTCATTCTC |
| *ASNS* | GGAAGACAGCCCCGATTTACT | AGCACGAACTGTTGTAATGTCA |
| *CHAC1* | GAACCCTGGTTACCTGGGC | CGCAGCAAGTATTCAAGGTTGT |
| *PPP1R15A* | ATGATGGCATGTATGGTGAGC | AACCTTGCAGTGTCCTTATCAG |
| *XBP1s* | CCCTCCAGAACATCTCCCCAT | ACATGACTGGGTCCAAGTTGT |
| *EDEM1* | CGGACGAGTACGAGAAGCG | CGTAGCCAAAGACGAACATGC |
| *HERPUD1* | ATGGAGTCCGAGACCGAAC | TTGGTGATCCAACAACAGCTT |
| *PDIA4* | GGCAGGCTGTAGACTACGAG | TTGGTCAACACAAGCGTGACT |
| *ACLY* | TCGGCCAAGGCAATTTCAGAG | CGAGCATACTTGAACCGATTCT |
| *SREBP1* | ACAGTGACTTCCCTGGCCTAT | GCATGGACGGGTACATCTTCAA |
| *18S* | GGCCCTGTAATTGGAATGAGTC | CCAAGATCCAACTACGAGCTT |

**Supplementary Table 4. Molecular docking parameters and predicted interface residues between OAS1 and FASN.**

| **Confidence Score** | **Docking Score** | **Interface Residues (FASN)** | **Interface Residues (OAS1)** |
| --- | --- | --- | --- |
| 0.9365 | -284.52 | **42**, 43, 45, **49**, 852, 854, 938, **940**, 941, 1694, 1702, 1705, 1717, 1718, 1720, 1721, **1722**, 1724, **1725**, **1726**, 1727, 1728, **1729**, 1730, **1731**, 1733, **1734**, **1735**, 1736, 1737, **1738**, **1739**, 1757, **1758**, **1781**, 1782, 1783, **1784** | 113-116, **140**, 160, 161, **162**, **163**, 164, 197, **200**, 201, 202, 238, 240, 241, **242**, 244, **245**, 246, **247**, 270, **271**, **272**, **273**, **274**, 277, 279, 346, **348**, 349, **351**, **353**, **399**, **400** |

*Residues within 5.0 Å distance at the interface are listed. Bold residues indicate strong interactions (< 3.0 Å).

*1725(FASN)-247(OAS1) (1.986 Å) and 1722(FASN) -247(OAS1) (2.269 Å) are the most potent interface residues, while 1734(FASN) is predicted interfacing with multiple sites including 242, 271, 272, 348(OAS1).
