# Supplementary material for: The OAS1-FASN axis promotes pancreatic cancer by coordinating lipogenic stress and the unfolded protein response
Source: Cell Death Dis. 2026 Jun 3;17(1):696. doi: 10.1038/s41419-026-08922-8 (PMC13448175; doi:10.1038/s41419-026-08922-8)
Supplement: Supplementary file 2 [file 41419_2026_8922_MOESM2_ESM.docx]

**Supplementary Figure 1. Pan-cancer expression and clustering QC for the OAS family.**

(A) Differential expression genes heatmap between FAM-high and FAM-low groups in the TCGA PAAD database.

(B) Consensus matrix heatmap (k=2) grouped according to the Fatty acid metabolism (FAM) hallmarks.

(C) Consensus CDF curves of the consensus index for k=2-9.

(D) Δ area under the CDF curve as a function of k.

(E) Kaplan-Meier overall survival for FAM-high vs. FAM-low in TCGA PAAD groups.

(F) Kaplan-Meier disease-free survival for FAM-high vs. FAM-low in PAAD groups.

(G) OAS family composite signature across TCGA cancer types and matched normal tissues.

(H) OAS1 expression across TCGA cancer types and matched normal tissues.

(I) Tumor vs. normal differential expression of OAS1/2/3/L by cancer type.

**Supplementary Figure 2. Distribution and expression of OAS family members in PDAC datasets.**

(A-C) OAS2 (A), OAS3 (B), and OASL (C) expression by z-score between normal and tumor tissues in three databases.

(D) Single-cell UMAPs of PDAC colored by OAS1/2/3/L expression in GSE11672.

(E) OAS1/2/3/L expression levels by cell type in PDAC of database GSE11672.

(F) Single-cell UMAPs of PDAC colored by OAS1/2/3/L expression in database CRA00160.

(G) OAS1/2/3/L expression levels by cell type in PDAC of database CRA001160.

**Supplementary Figure 3. Blocking IFN signaling does not affect proliferation and lipid metabolism in PDAC cells.**

(A-B) qRT-PCR of interferon-stimulated genes after IFN-α treatment (±α-IFNAR2) of Patu-8988 (A) and PDC0034 (B).

(C-D) CCK-8 proliferation assay with or without IFNAR2 neutralization of Patu-8988 (C) and PDC0034 (D).

(E) FASN expression by IF staining with or without IFNAR2 neutralization of Patu-8988 (scale bar: 50μm).

(F) BODIPY lipid staining and with or without IFNAR2 neutralization of Patu-8988 (scale bar: 50μm).

(G) Relative FASN functional output in Patu-8988 with or without IFNAR2 neutralization.

For IF staining in (E), primary antibodies were diluted as specified in Supplementary Table 1 (OAS1 1:200, FASN 1:100) and incubated overnight at 4℃ followed by either FITC-conjugated Goat anti-rabbit IgG (1:100) or Cy3-conjugated Goat anti-rabbit IgG (1:100) incubation for 1 hour at room temperature. BODIPY (F) was incubated at room temperature for 20 min.

**Supplementary Figure 4. OAS1 knockdown inhibits lipid metabolism and ER stress in PDAC cells.**

(A) Principal Component Analysis (PCA) for untargeted metabolomics.

(B) Orthogonal Partial Least Squares Discriminant Analysis (OPLS-DA) for untargeted metabolomics.

(C) qRT-PCR of ER-stress/ATF4-pathway genes verification.

(D) IF staining for BiP and ATF4 with DAPI of differently treated Patu-8988 (scale bar: 20μm).

(E) qRT-PCR of representative genes of three ER-stress-related pathways by Tunicamycin and ER-pathway inhibitor treatments as indicated.

(F) Confocal ER Tracker imaging of Tunicamycin and ER-pathway inhibitor treatments as indicated of Patu-8988 (scale bar: 20μm).

(G) BODIPY lipid staining, and OAS1 and p-AMPK/FASN/SCD1/BiP/ATF4 IF staining images of PDC0034 with DAPI (shNC vs. shOAS1) (scale bar: 20μm).

(H) Live-cell imaging of lipid droplets and confocal ER Tracker staining images of PDC0034 (shNC vs. shOAS1) (Yellow arrow: lipid droplets; scale bar: 20μm).

(I) qRT-PCR of lipid-metabolism and ER-stress genes after OAS1 knockdown of Patu-8988.

For IF staining in (D) and (G), primary antibodies were diluted as specified in Supplementary Table 1 (OAS1 1:200, FASN 1:100, p-AMPK 1:200, SCD1 1:100, BiP 1:100, ATF4 1:200) and incubated overnight at 4℃ followed by either FITC-conjugated Goat anti-rabbit IgG (1:100) or Cy3-conjugated Goat anti-rabbit IgG (1:100) incubation for 1 hour at room temperature. ER tracker (F) and BODIPY (G) were incubated at room temperature for 20 min.

**Supplementary Figure 5.** **The Core domain but not enzymatic activity of OAS1 is essential for its interaction with FASN and promotion of PDAC progression.**

(A) qRT-PCR analysis of OAS1 mRNA expression in Patu-8988 and PDC0034 cells transfected with indicated OAS1 variants.

(B) CCK-8 assay showing the proliferation of Patu-8988 and PDC0034 cells after transfection with Vector, WT, Mut, or CT constructs.

(C) Representative images and quantification of Transwell migration assays in the indicated groups of Patu-8988 and PDC0034 (scale bar: 200μm).

(D) Relative FASN functional output in the indicated groups of Patu-8988 and PDC0034.

(E) Representative BODIPY staining images showing lipid droplet accumulation (scale bar: 20μm).

BODIPY (E) was incubated at room temperature for 20 min.

**Supplementary Figure 6. OAS1 interacts with FASN and increases lipid load in PDAC cells.**

(A-B) Western blotting of FASN expression (A) and gray densitometry heatmap (B) of Vector vs. FASN-OE groups of Patu-8988 and PDC0034.

qRT-PCR analysis of OAS1 mRNA expression in Patu-8988 and PDC0034 cells transfected with indicated OAS1 variants.

(C) BODIPY lipid staining of Vector vs. FASN-OE groups of Patu-8988 and PDC0034 (scale bar: 20μm).

(D-E) Overall survival (D) and Disease-free survival (E) by FASN grouping in the TCGA PAAD database.

(F) FASN expression by z-score between normal and tumor tissues in three databases.

(G) qRT-PCR validation of FASN rescue in Patu-8988 and PDC0034.

(H) Gray densitometry heatmap of western blotting across indicated groups of Patu-8988 and PDC0034.

(I) CCK-8 proliferation assay across indicated groups of FASN-rescue of Patu-8988 and PDC0034.

(J) Wound-healing assay quantification across indicated groups of FASN-rescue of Patu-8988 and PDC0034.

(K) Live-cell lipid-droplet imaging across indicated groups of FASN-rescue of PDC0034 (Yellow arrow: lipid droplets; scale bar: 20μm).

(L) qRT-PCR of lipid- and ER-related genes across indicated groups of FASN-rescue of Patu-8988.

(M-N) BODIPY lipid staining (M) and IF staining (N) for OAS1 and FASN of PDC0034 with DAPI (indicated groups of FASN-rescue) (scale bar: 20μm).

BODIPY (M) was incubated at room temperature for 20 min. For IF staining in (N), primary antibodies were diluted as specified in Supplementary Table 1 (OAS1 1:200, FASN 1:100) and incubated overnight at 4℃ followed by either FITC-conjugated Goat anti-rabbit IgG (1:100) or Cy3-conjugated Goat anti-rabbit IgG (1:100) incubation for 1 hour at room temperature.

**Supplementary Figure 7. OAS1-FASN-driven lipid overload engages an ATF4-mediated adaptive ER stress response in PDAC cells.**

(A) Live-cell dual imaging of ER Tracker and lipid droplets across indicated groups of Patu-8988 based on OAS1 knockdown and Tunicamycin treatment (Yellow arrow: lipid droplets; scale bar: 20μm).

(B) BODIPY lipid staining and ER Tracker staining of Patu-8988 based on OAS1 knockdown and Tunicamycin treatment (scale bar: 20μm).

(C) qRT-PCR of lipid-metabolism and ER-stress genes mRNA expression of Patu-8988 after Tunicamycin treatments.

(D) BODIPY lipid staining and BiP-ATF4 IF staining images of indicated groups of PDC0034 based on OAS1 knockdown and Tunicamycin treatment with DAPI (scale bar: 20μm).

(E) Gray densitometry heatmap of BiP and OAS1 expression across indicated groups of Patu-8988 and PDC0034.

(F-G) qRT-PCR analysis of OAS1 (F) and ATF4 (G) mRNA expression in Patu-8988 and PDC0034 cells transfected with shNC/shOAS1 and siNC/siATF4. (^*^ compared to group shNC+siNC, ^#^ compared to group shOAS1+siNC.)

(H) CCK-8 assay of the indicated groups of Patu-8988 and PDC0034 based on ATF4 knockdown upon OAS1 deficiency.

(I-J) Representative images of colony formation assay (I) and Transwell assay (J) of PDC0034 based on ATF4 knockdown upon OAS1 deficiency (scale bar: 200μm).

ER tracker (A-B) and BODIPY (B) were incubated at room temperature for 20 min. For IF staining in (D), primary antibodies were diluted as specified in Supplementary Table 1 (OAS1 1:200, BiP 1:100, ATF4 1:200) and incubated overnight at 4℃ followed by either FITC-conjugated Goat anti-rabbit IgG (1:100) or Cy3-conjugated Goat anti-rabbit IgG (1:100) incubation for 1 hour at room temperature.

**Supplementary Figure 8. Pharmacological inhibition of FASN by TVB-2640 suppresses PDAC progression and synergizes with OAS1 knockdown.**

(A) Immunofluorescence staining of ATF4, ER-Tracker and BODIPY in PDC0034 cells across the experimental groups based on OAS1 and ATF4 knockdown (scale bar: 20μm).

(B-C) Dose-response curves and IC_50_ values of TVB-2640 in Patu-8988 (B) and PDC0034 (C) cells based on OAS1 knockdown. Cell viability was measured by CCK-8 assay after 72 hours of treatment.

(D) Representative images and statistical analysis of colony formation assays in Patu-8988 and PDC0034 cells treated with DMSO or TVB-2640 following shNC or shOAS1 transfection.

(E) Representative images and statistical analysis of Transwell assays in Patu-8988 and PDC0034 cells treated with DMSO or TVB-2640 following shNC or shOAS1 transfection.

(F) BODIPY lipid staining, ER Tracker staining, and BiP-ATF4 IF staining images of indicated groups of PDC0034 based on OAS1 knockdown and TVB-2640 treatment with DAPI (scale bar: 20μm).

ER tracker (A, F) and BODIPY (A) were incubated at room temperature for 20 min. For IF staining in (A) and (F), primary antibodies were diluted as specified in Supplementary Table 1 (BiP 1:100, ATF4 1:200) and incubated overnight at 4℃ followed by either FITC-conjugated Goat anti-rabbit IgG (1:100) or Cy3-conjugated Goat anti-rabbit IgG (1:100) incubation for 1 hour at room temperature.

**Supplementary Figure 9. OAS1-FASN axis promotes PDAC progression and lipid metabolism in vivo.**

(A) Representative IHC staining for Ki-67, OAS1, PLIN2, FASN, BiP of Patu-8988 subcutaneous xenografts (scale bar: 200μm).

(B) Gross liver images of KPC-1199 orthotopic transplantation models collected at endpoint (Yellow arrow: metastatic lesion; n=7).

(C) Representative IHC staining for N-cadherin, OAS1, PLIN2, FASN, BiP of KPC-1199 orthotopic transplantation models’ liver metastases (scale bar: 200μm).

For IHC staining in (A) and (C), primary antibodies were diluted as specified in Supplementary Table 1 (Ki-67 1:1000, OAS1 1:200, PLIN2 1:200, FASN 1:1000, BiP 1:100, N-cadherin 1:2000) and incubated overnight at 4℃ followed by HRP-conjugated secondary antibodies incubation for 1 hour at room temperature. Visual signal was developed using DAB, and the sections were counterstained with hematoxylin for nuclear visualization.
